# Supplementary material for: Auto-expansion of in vivo HDAd-transduced hematopoietic stem cells by constitutive expression of tHMGA2
Source: Mol Ther Methods Clin Dev. 2024 Aug 13;32(3):101319. doi: 10.1016/j.omtm.2024.101319 (PMC11399618; doi:10.1016/j.omtm.2024.101319)
Supplement: Document S1. Figures S1–S6 and Tables S1 and S3 [file mmc1.pdf]

OMTM, Volume *32*

Supplemental information

Auto-expansion of *in vivo* HDAd-transduced hematopoietic stem cells by constitutive expression of tHMGA2

Hongjie Wang, Aphrodite Georgakopoulou, Evangelos Nizamis, Ka Wai Mok, Raïssa Eluère, Robert A. Policastro, Paul N. Valdmanis, and André Lieber

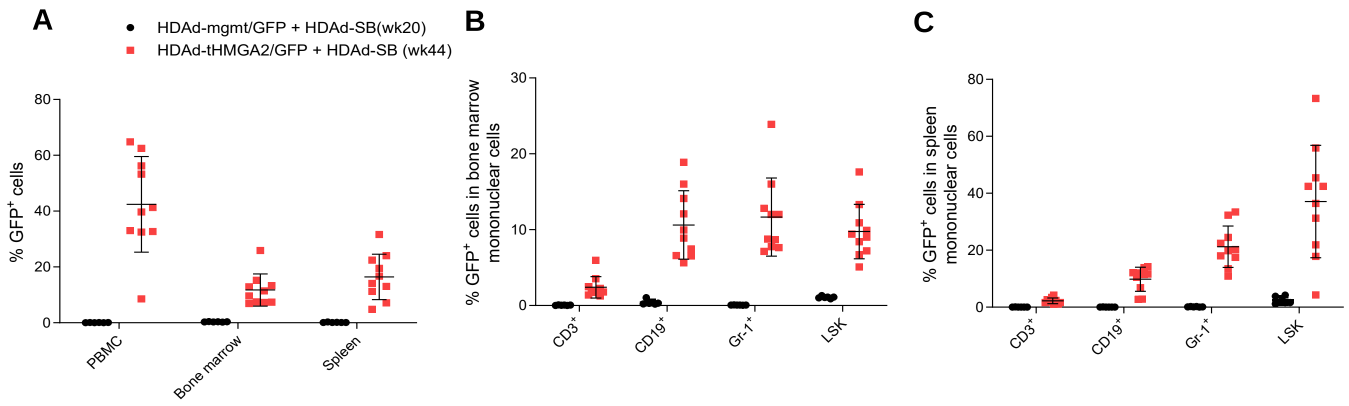

Fig.S1. ***In vivo*** **transduction with HDAd-mgmt/GFP + HDAd-SB and HDAd-tHMGA2/GFP + HDAd-SB- Analysis of PBMC, bone marrow, and spleen cells. A)** The percentage of GFP-positive cells in total PBMC, bone marrow and spleen mononuclear cells. **B) and C)** Percentage of GFP-positive within lineage-positive cells and LSK cells within bone marrow and spleen. Each symbol is an individual animal. Mean and error bars (+/-SEM) are shown.

A

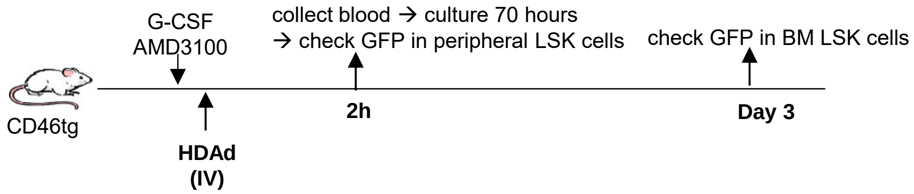

B

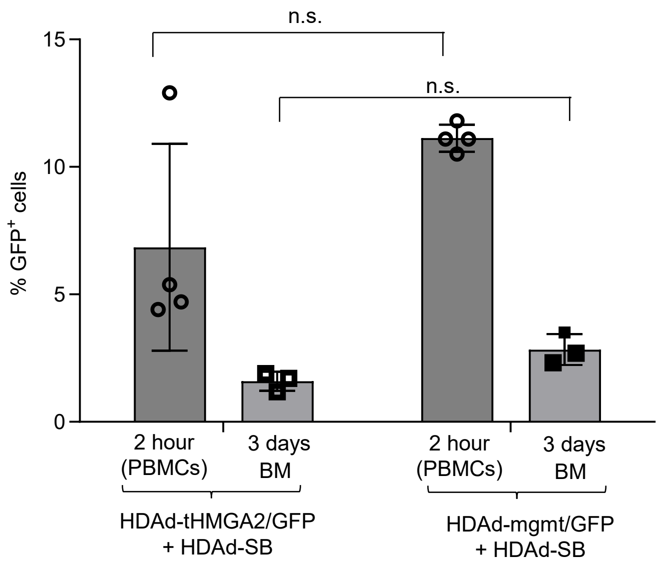

Fig.S2. Initial *in vivo* transduction of mobilized HSCs. A) Schematic of experiment. HSCs in hCD46tg mice were mobilized by G-CSF for 4 days, followed by a single injection of AMD3100 on day 5. Thirty and 60 minutes after AMD3100, animals were intravenously injected with HDAd vectors through the retro-orbital plexus (4×10^{10} viral particles per injection per mouse; same dose as in Fig.1). Transduction of mobilized, peripheral LSK cells was analyzed by harvesting PBMCs at 2 hours after the last virus injection, and culturing them for 3 days to allow for GFP expression. At day after HDAd injection, animals were sacrificed and the percentage of GFP^{+} LSK cells in the bone marrow was measured. **B)** Initial transduction of LSK cells. Shown are percentages of GFP^{+} LSK cells after injection of HDAd-tHMGA2/GFP + HDAd-SB and HDAd-mgmt/GFP + HDAd-SB in (mobilized) peripheral blood LSK cells (at 2 hours) and in LSK cells that returned to the bone marrow (day 3). Each symbol is an individual animal.

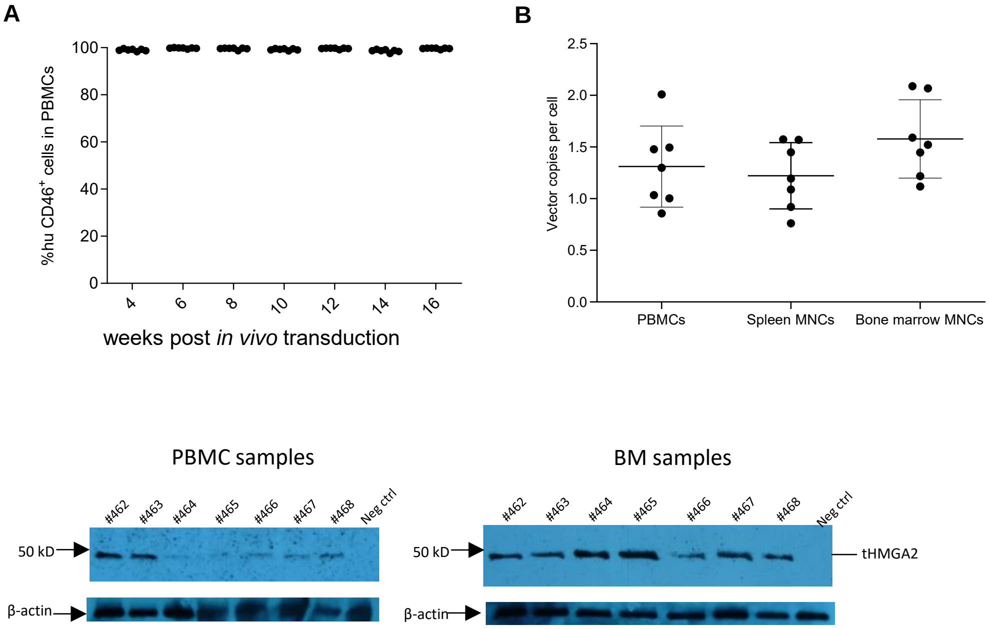

Fig.S3 Analysis of secondary recipients transplanted with Lin$^-$ cells from HDAd-tHMGA2+HDAdSB transduced mice. A) Engraftment based on the percentage of human CD46-positive cells in PBMCs. **B)** VCN per cell in PBMCs, BM MNCs and spleen MNCs of secondary recipients at week 18 after transplantation. Data are shown as means ± SEM. **C)** Western blot showing the expression of tHMGA2 protein in PBMCs and bone marrow MNCs at week 18 after transplantation.

A

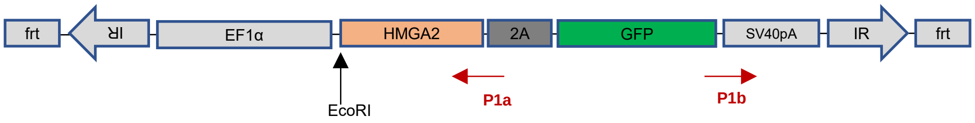

B

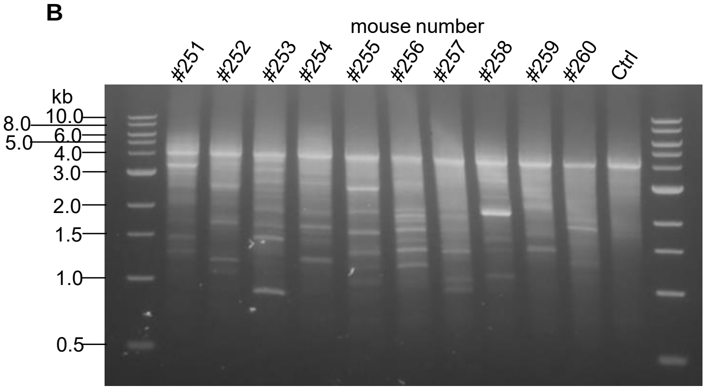

Fig.S4 Integration analysis by inverse PCR. A) The transposon (between two IR elements) in the HDAd vectors are integrated into chromosomal DNA. Genomic DNA was digested with EcoRI, which cuts inside the transposon and re-ligated. PCR were performed with two vector specific primers (P1a, P1b)to amplify transposon/chromosomal DNA junctions. **B)** Agarose gel electrophoresis showing PCR products. Note that the vector specific primer set used has an unspecific background band (~4.0 kb) in all mice including untransduced control mice.

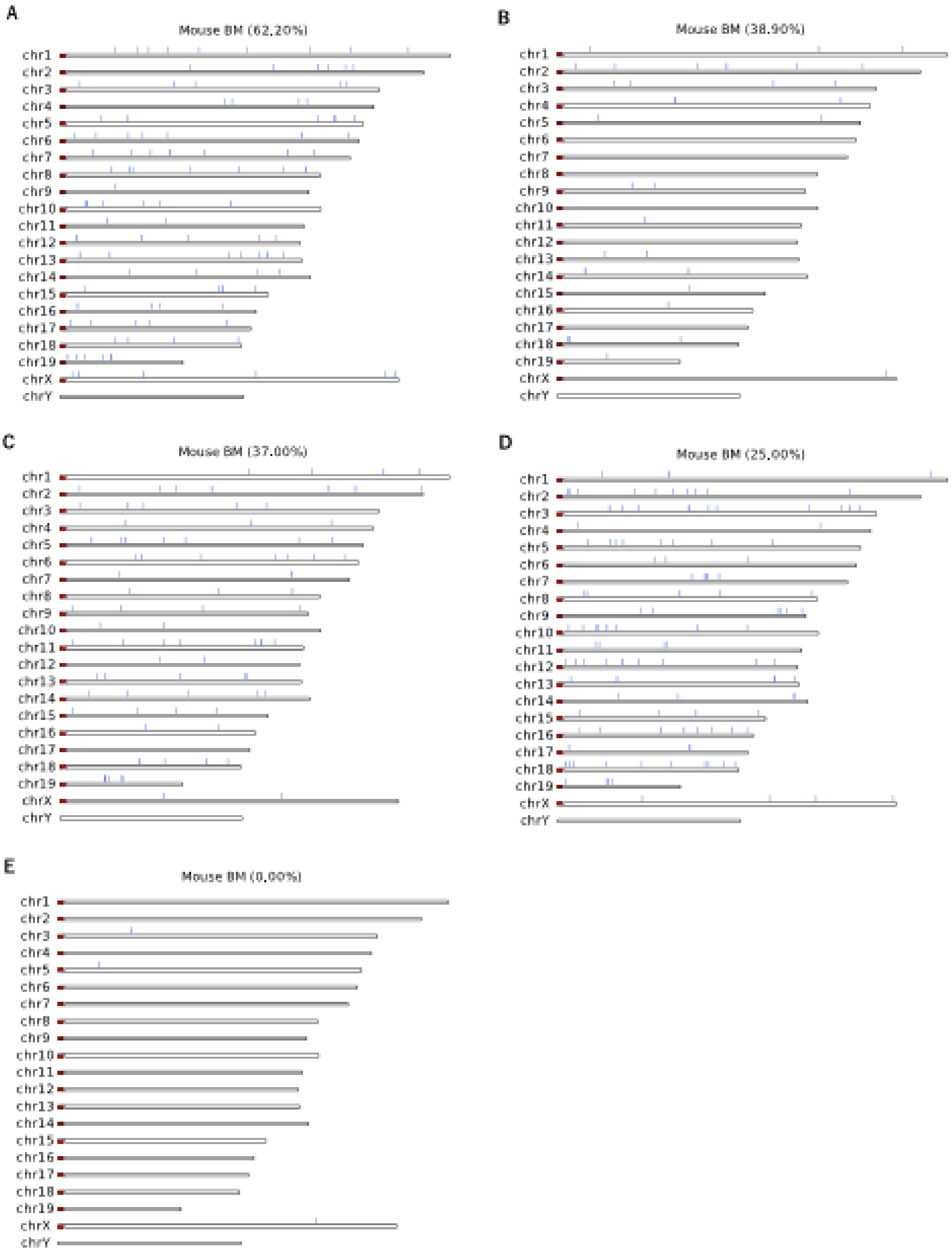

Fig.S5 Chromosomal distribution of integration sites. Chr

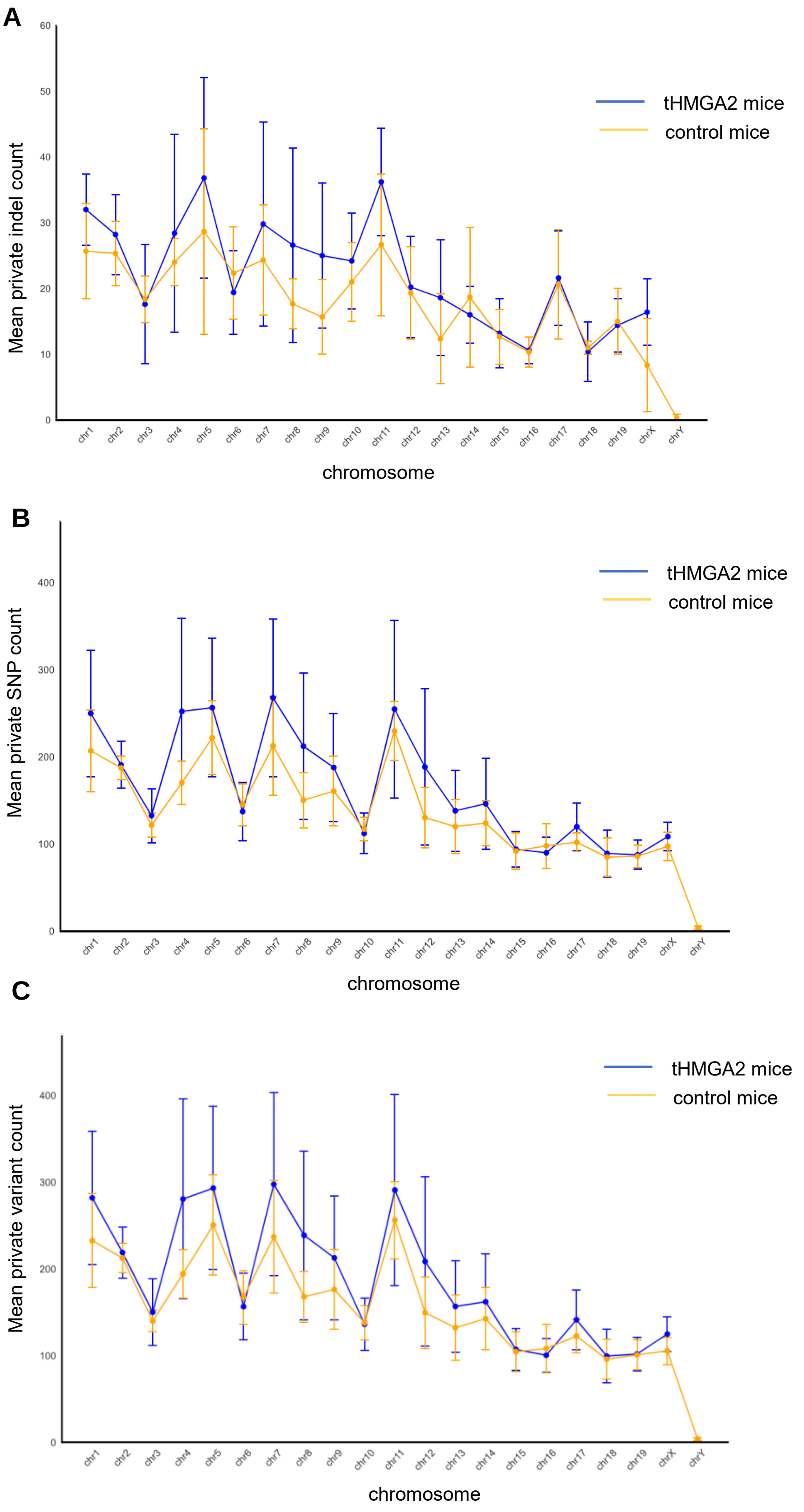

Fig.S6 Lineplot with error bars showing standard deviation of values for private variants of HDAd-tHMGA2 + HDAd-SB compared with untreated control mice. Private variants (SNPs/Indels) were categorized by chromosome and compared between HDAd-tHMGA2 + HDAd-SB treated mice (BM1-BM-5) compared with untreated control mice (N1-N3). Mean values and standard deviations were calculated across samples for each chromosome. Private variants are unique to each sample, meaning they are not found in any other treated or control samples. They were calculated for **A)** All Variants**, B)** Indels, and **C)** SNPs.

TableS1 TRACE sequencing primers. The oligo sequences used for tagmentation and PCR in TRACE sequencing.

Oligo	Sequence
Tn5 adaptor top	GACCCGGGAGATCTGAATTCAGTGGCACAGCAGTTAGGNNNNNNNNAGATGTGTATAAGAGACAG
Tn5 adaptor bottom	Phos-CTGTCTCTTATACACATC/invT
Tn1c (PCR #1 F)	CTTGTGTCATGCACAAAGTAGATGTCC
LCI (PCR #1 R)	GACCCGGGAGATCTGAATTC
MA-F (PCR #2 F)	ACACTCTTTCCCTACACGACGCTCTTCCGATCTNNNNCGAGTTTTAATGACTCCAACT
MA-LCII (PCR #2 R)	GTGACTGGAGTTCAGACGTGTGCTCTTCCGATCTAGTGGCACAGCAGTTAGG

TableS2 Genome-wide insertion locations (provided as Excel file)

TableS3 Statistics of the coverage on target exon regions

[Target] Target Reads	237195756
[Target] Target Data (Mb)	27023.94
[Target] Len of region	49387927
[Target] Average depth	547.18
[Target] Coverage (>0x)	99.90%
[Target] Coverage (>/=10x)	99.61%
[Target] Coverage (>/=30x)	98.72%
[Target] Coverage (>/=100x)	94.34%
[flank] flank size	200
[flank] Len of region	118267836
[flank] Average depth	318.75
[flank] flank reads	254868740
[flank] flank Data (Mb)	37697.90
[flank] Coverage (>0x)	99.53%
[flank] Coverage (>/=10x)	95.37%
[flank] Coverage (>/=30x)	85.96%
[flank] Coverage (>/=100x)	66.71%
