# Supplementary material for: Auto-expansion of in vivo HDAd-transduced hematopoietic stem cells by constitutive expression of tHMGA2
Source: Mol Ther Methods Clin Dev. 2024 Aug 13;32(3):101319. doi: 10.1016/j.omtm.2024.101319 (PMC11399618; doi:10.1016/j.omtm.2024.101319)
Supplement: Document S2. Article plus supplemental information [file mmc3.pdf]

Molecular Therapy
Methods & Clinical Development
Original Article

Auto-expansion of *in vivo* HDAd-transduced hematopoietic stem cells by constitutive expression of tHMGA2

Hongjie Wang,[1] Aphrodite Georgakopoulou,[1] Evangelos Nizamis,[1] Ka Wai Mok,[2] Raïssa Eluère,[2] Robert A. Policastro,[2] Paul N. Valdmanis,[1] and André Lieber[1,3]

[1]University of Washington, Department of Medicine, Division of Medical Genetics, Seattle, WA 98195, USA; [2]Ensoma, Inc., Boston, MA, USA; [3]University of Washington, Department of Laboratory Medicine and Pathology, Seattle, WA 98195, USA

We developed an *in vivo* hematopoietic stem cell (HSC) gene therapy approach that does not require cell transplantation. To achieve therapeutically relevant numbers of corrected cells, we constructed HSC-tropic HDAd5/35++ vectors expressing a 3′ UTR truncated HMGA2 gene and a GFP reporter gene. A SB100x transposase vector mediated random integration of the tHMGA2/GFP transgene cassette. HSCs in mice were mobilized by subcutaneous injections of G-CSF and AMD3100/Plerixafor and intravenously injected with the integrating tHMGA2/GFP vector. This resulted in a slow but progressive, competitive expansion of GFP$^+$ PBMCs, reaching about 50% by week 44 with further expansion in secondary recipients. Expansion occurred at the level of HSCs as well as at the levels of myeloid, lymphoid, and erythroid progenitors within the bone marrow and spleen. Importantly, based on genome-wide integration site analyses, expansion was polyclonal, without any signs of clonal dominance. Whole-exome sequencing did not show significant differences in the genomic instability indices between tHGMGA2/GFP mice and untreated control mice. Auto-expansion by tHMGA2 was validated in humanized mice. This is the first demonstration that simple injections of mobilization drugs and HDAd vectors can trigger auto-expansion of *in vivo* transduced HSCs resulting in transgene-marking rates that, theoretically, are curative for hemoglobinopathies.

INTRODUCTION

In vivo HSC gene therapy

Despite encouraging preclinical and clinical results, current *ex vivo* HSC gene therapy protocols have multiple shortcomings throughout the process: (1) harvesting HSCs by leukapheresis or bone marrow (BM) aspiration (invasive procedures), (2) myeloablation by chemotherapy (high-dose chemotherapy-related side effects, infectious disease complications, conditioning-associated genotoxicity), (3) *in vitro* HSC culture and transplantation (loss of HSC pluripotency during extended *ex vivo* culture, need for specialized facility/staff), and (4) the cost of the approach. Because of the cost and technical complexity, it is unlikely that *ex vivo* protocols will be widely applicable. We have developed a minimally invasive and readily translatable approach for *in vivo* HSC gene therapy of hemoglobinopathies. It involves the mobilization of HSCs from the BM into the peripheral blood and a single intravenous (i.v.) injection of helper-dependent adenovirus vectors (HDAd) that target receptors present on HSCs. A large fraction of mobilized HSCs transduced in peripheral circulation return to the BM and spleen and persist there long term. Based on GFP expression in HSCs analyzed in the BM 7 days after HDAd injection, transduction rates are 15%–20% in mouse HSCs (lineage-negative Sca1$^+$/cKit$^+$ [LSK] cells)[1,2] and ~7% in rhesus HSCs (CD34$^+$/CD45RA$^-$/CD90$^-$).[3] However, not every transduced HSC is stably modified with integrated transgenes or permanent genome edits. Our currently used *Sleeping Beauty* transposase integrating system requires co-infection of two vectors, the transposon vector and the SB100x vector for stable expression, which limits the number of stably transduced HSCs.[4,5] For genome editors (CRISPR-Cas9, base editors, prime editors), even though cells are transduced, target site editing requires a certain expression level (or vector copy number [VCN])]and target site accessibility, which can depend on the proliferation/differentiation stage of cells.[6–8] Based on previous work, we estimate that less than 1% of HSC have stably integrated transgenes or permanent, therapeutic genomic edits after *in vivo* HSC transduction.[4,5,9,10] These correction levels would be sufficient to phenotypically cure X-linked severe combined immunodeficiency (SCID) or adenosine deaminase-deficient SCID (based on HSC transplantation studies),[11] but they would not be sufficient for hemoglobinopathies (β-thalassemia and sickle cell disease [SCD]), which requires at least 20% of therapeutically corrected erythroid cells in the periphery.[12] To achieve therapeutically relevant gene correction levels, we have used *in vivo* expansion of transduced HSCs. One such approach is based on a mutant O^6-methylguanine-DNA methyltransferase (mgmt^{P140K}) gene that confers resistance to O^6-benzylguanine (O^{6}BG)/bis-chloroethylnitrosourea (BCNU/Carmustine).[13] We tested this approach in mice[14]

Received 15 April 2024; accepted 8 August 2024;
https://doi.org/10.1016/j.omtm.2024.101319.

Correspondence: André Lieber, University of Washington, 1705 NE Pacific St, Seattle, WA 98195, USA.
E-mail: lieber00@uw.edu

© 2024 The Author(s). Published by Elsevier Inc. on behalf of The American Society of Gene and Cell Therapy.
This is an open access article under the CC BY license (http://creativecommons.org/licenses/by/4.0/).

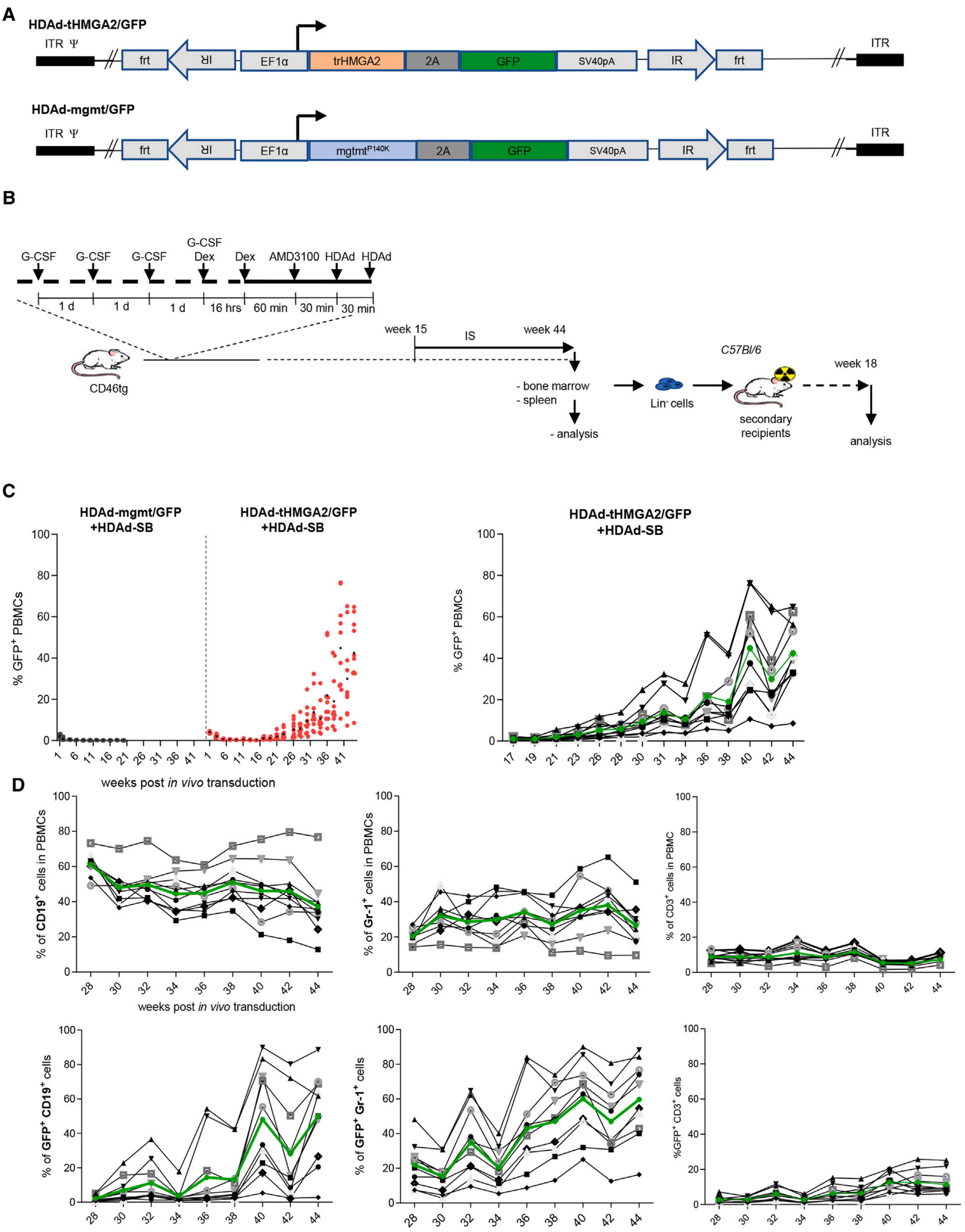

(legend on next page)

and non-human primates (NHPs)[15–17] using three to four injections of O^{6}BG and BCNU at doses that were lower than used for chemotherapy of cancer. In studies toward *in vivo* HSC gene therapy of hemoglobinopathies, we used O^{6}BG/BCNU selection in combination with *in vivo* γ-globin gene addition,[18] *in vivo* base editing to reactivate γ-globin,[9] and *in vivo* prime editing to correct the SCD mutation.[10]

However, for non-oncological applications, including genetic and infectious diseases, an *in vivo* selection approach that avoids treatment with cyto- and/or genotoxic agents (or any pharmacological intervention at all) would be preferable. We have therefore been exploring a series of alternative *in vivo* HSC selection/expansion methods. One of these strategies is based on the constitutive expression of a truncated High-Mobility Group AT-hook 2 (tHMGA2) gene. The full-length HMGA2 gene encodes a small protein with three DNA-binding AT-hook domains that modulates chromatin structure, transcription, and epigenetic regulation of multiple genes.[19] It plays crucial roles in the proliferation, cell-cycle progression, and self-renewal of HSCs.[20] Transgenic mice that expressed 3′ UTR truncated human HMGA2 in HSCs showed proliferative hematopoiesis with increased numbers in all lineages of peripheral blood cells. HSCs from these mice had a growth advantage after serial transplantation.[21] In a recent *ex vivo* HSC gene therapy study in rhesus macaques,[22] CD34$^+$ cells were transduced with a lentivirus vector to constitutively over-express the 3′ UTR truncated HMGA2 under control of the MSCV promoter. After transplantation into lethally irradiated animals, these cells demonstrated increased self-renewal potential that did not result in any hematological malignancies.

Here, we show in mice that, after *in vivo* HSC transduction, the expression of tHMGA2 from an SB100x-integrated transgene cassette results in expansion of HSCs and progenitors without clonal dominance. GFP marking levels reached over 50% in all lineages in the majority of mice.

RESULTS

HDAd vectors

For *in vivo* HSC transduction, we used integrating HDAd5/35++ vectors.[23,24] These vectors are derived from Ad5 but target CD46 through Ad35 fibers. The affinity of Ad35 fiber knobs to CD46 was enhanced by a series of mutations.[1] CD46 is expressed at high levels on primitive human and monkey HSCs and at lower levels on other nucleated cells.[2,16] CD46 expression on non-HSCs is not sufficient to mediate HDAd5/35++ transduction.[3,16,25] CD46 transgenic mice contain the human CD46 locus and express human CD46 in a pattern similar to humans, including high-level expression on HSCs.[4,26] We employed two HDAd5/35++ vectors that had an identical payload structure except for the key transgene (Figure 1A). HDAd-tHMGA2/GFP contained an expression cassette linking the 3′ UTR-truncated human HMGA (tHMGA2) gene[22] with the GFP reporter gene through a self-splicing picornavirus 2A peptide. HDAd-mgtm/GFP contained the mgmt^{P140K} mutant gene instead of the tHMGA2 gene. Transgene transcription was under the control of the ubiquitously active EF1α promoter (allowing for expression in all blood cell lineages). Transgene integration was mediated by a hyperactive *Sleeping Beauty* transposase (SB100x) system.[27] SB100x is expressed from a second HDAd5/35++ vector (HDAd-SB) that is co-administered with the tHMGA2/GFP or mgmt/GFP transposon containing vector.

Expansion of tHMGA2/GFP-transduced hematopoietic stem and progenitor cells in mice

CD46 transgenic mice were mobilized by subcutaneous (s.c.) injections of human recombinant G-CSF (250 μg/kg/mouse/day, 4 days) followed by an s.c. injection of AMD3100 (5 mg/kg) on day 5. In addition, animals received dexamethasone (10 mg/kg, intraperitoneally [i.p.]) 16 and 2 h before virus injection to blunt innate toxicity associated with i.v. HDAd injection. Thirty and 60 min after AMD3100, animals were intravenously injected with HDAd vectors through the retro-orbital plexus (4×10^{10} viral particles per injection per mouse) (Figure 1B). Group 1 (N = 10) were injected with HDAd-mgmt/GFP + HDAd-SB. Group 2 (N = 10) received HDAd-tHMGA2/GFP + HDAd-SB. Blood samples were taken every other week for a total of 44 weeks. There were no unexpected/adverse side effects in the treated mice during the observation period. Peripheral blood mononuclear cells (PBMCs) and blood cell lineages were analyzed for GFP expression by flow cytometry (Figure 1C). Twenty weeks after HDAd-tHMGA2/GFP + HDAd-SB injection, the percentage of GFP$^+$ PBMCs started increasing above the background of 1% in all the mice reaching an average of 50% (range 10%–70%) by week 44. In mice of group 1 that received HDAd-mgmt/GFP + HDAd-SB, GFP marking in PBMCs was steady at </ = 1% Notably, mice in both groups did not receive O^{6}BG/BCNU treatment; however, they were given immunosuppressive drugs to inhibit potential B and

Figure 1. *In vivo* HSC transduction—analysis of peripheral blood cells

(A) HDAd vector structures. Transgene transcription was under the control of the ubiquitously active EF1a promoter. The MGMT^{p140k} gene was replaced by 3′ UTR truncated tHMGA2 gene. (B) Schematic of the experiment. *In vivo* transduction of mobilized hCD46tg mice. HSCs were mobilized by s.c. injections of human recombinant G-CSF for 4 days followed by one s.c. injection of AMD3100. Thirty and 60 min after AMD3100 injection, animals were injected i.v. with HDAd-tHMGA2/GFP + HDAd-SB (1:1 mixture) or HDAd-mgmt/GFP + HDAd-SB (1:1) (two injections, each 4×10^{10} viral particles). Mice were treated with immunosuppressive drugs to avoid immune responses against the human tHMGA2 protein. Mice were followed until week 44, when animals were sacrificed for analysis. BM lineage-negative (Lin$^-$ cells) were transplanted into lethally irradiated C57Bl/6 mice, which were then followed for 18 weeks. (C) Percentage of GFP$^+$ PBMCs in primary (*in vivo* transduced) mice. Each symbol is an individual animal. Control group (HDAd-mgmt/GFP + HDAd-SM) was followed up to week 20. The HDAd-tHMGA2/GFP + HDAd-SB) group was followed up to week 44. The black bars in this group are the average percentages of GFP$^+$ PBMCs in the 10 mice for each time point. The right panel shows the percentage of GFP$^+$ PBMCs starting from week 17. The curve in green represents the average. (D) Percentage of lineage-positive cells within PBMCs and percentage of GFP$^+$ cells within lineage-positive PBMCs analyzed from weeks 28 to 44 post *in vivo* transduction with HDAd-tHMGA2/GFP + HDAd-SB. The curves in green represent the average percentage. Each symbol represents an individual mouse.

T cell responses against human tHMGA2. Notably, in previous studies, we have followed *in vivo* HSC-transduced CD46tg mice without O^6BG/BCNU selection for 30 weeks.[14] GFP marking in PBMCs was less than 1% in these studies. We therefore ended this study in the HDAd-mgmt/GFP + HDAd-SB cohort earlier at 20 weeks because no increase in GFP^+ PBMCs was expected anymore.

In peripheral blood, the percentage of $CD19^+$ (B cells), Gr-1^+ (granulocytes), and $CD3^+$ cells (T cells) did not significantly change from weeks 28 to 44 (the end of the study) (Figure 1D, upper panel). However, the percentage of GFP^+ cells in these fractions increased, in $CD19^+$ cells from 2% to 50% (on average), in $Gr1^+$ cells from 20% to 60%, and in $CD3^+$ cells from 2% to 10% (Figure 1D, lower panel). This indicates a preferential expansion of transduced (tHMGA2-expressing) cells along with a compensatory reduction in the generation of untransduced white blood cell progenitors.

Further analyses included BM and spleen mononuclear cells (MNCs) at the endpoints of the study (Figure 2). The fraction of lineage-positive $CD3^+$, $CD19^+$, and $Gr1^+$ cells in all MNCs was similar in both the HDAd-mgmt/GFP + HDAd-SB and HDAd-tHMGA2 + HDAd-SB cohorts (Figures 2A and 2B). However, while the percentage of GFP^+ cells in lineages was less than 1% in BM and spleen of HDAd-mgmt/GFP + HDAd-SB-transduced mice (Figure S2), an average of ~3% $GFP^+/CD3^+$ cells, 10% $GFP^+/CD19^+$, and 10% $GFP^+/Gr1^+$ cells were found in the BM of HDAd-tHMGA2 + HDAd-SB-injected animals (Figure 2C, BM). In the spleen, an average of ~3.5% $GFP^+/CD3^+$ cells, 10% $GFP^+/CD19^+$, and 20% $GFP^+/Gr1^+$ cells were found (Figure 2C, Spleen). The average percentage of GFP^+ cells in PBMCs and lineages was ~4-fold higher than in the BM and spleen (Figure 2C). This indicates that tHMGA2-mediated expansion occurred after the cells exited the BM. We speculate that this involves the extramedullary expansion of immune cells (NK, T, B cells, etc.), perhaps stimulated by the human tHMGA2 that is expressed after *in vivo* transduction. The differences in GFP^+ MNCs in the BM and spleen were not significant. While flow analysis of denucleated erythroid cells (e.g., erythrocytes) is difficult, analysis of erythroid progenitors in the BM showed on average 16% of $GFP^+/Ter119^+$ cells (Figure 2D, $Ter119^+$). GFP marking rates were not significantly different in $CD19^+$ and Gr-1^+ lineages, however, they were about 4-fold lower in $CD3^+$ cells (Figure 2D). This indicates that tHMGA2-mediated expansion occurred in all (myeloid, lymphoid, and erythroid) lineages, whereby expansion of $CD3^+$ cells was less pronounced. Within BM MNCs, about 10% of LSK cells, a cell fraction that is enriched for HSCs, were GFP^+ (Figure 2D, LSK). This is 10-fold higher than in mice that received the HDAd-mgmt/GFP + HDAd-SB control vector (Figure S1), suggesting an expansion at the level of HSCs as well. In the spleen, on average 35% of LSK cells were GFP^+ in HDAd-tHMGA2/GFP mice (Figure 2E). The significantly higher percentage of GFP^+ LSK cells in the spleen vs. BM could be due to a more efficient return of mobilized and transduced HSCs to the spleen.[4] The average percentage of GFP^+ cells in splenic $CD3^+$, $CD19^+$, and Gr-1^+ cells was 3%, 10%, and 25%, respectively.

Expansion of GFP^+ cells in all lineages was also supported by the analysis of progenitor colonies that formed after plating of lineage-negative (Lin^-)) BM cells isolated at week 44 after HDAd-tHMGA2 + HDAd-SB, GFP^+ colonies included CFU-GEMM-, CFU-G-, BFU-E-, and CFU-E-expressing GFP (Figure 2F). On average about 15% of colonies were GFP^+ in individual mice.

To exclude that the differences in GFP marking rates of PBMCs at week 44 are due to a more efficient initial *in vivo* HSC transduction by HDAd-tHMGA2/GFP + HDAd-SB (vs. HDAd-mgmt/GFP + HDAd-SB), we performed an additional *in vivo* experiment (Figure S2A). CD46tg mice were mobilized and intravenously HDAd injected as described for Figure 1. Two hours after HDAd injection, blood samples were collected and PBMCs were cultured for 3 days to allow for GFP expression, which was measured by flow cytometry in LSK cells (Figure S2B). The analysis reflects the initial transduced of mobilized HSCs in the periphery. It was on average 7% for HDAd-tHMGA2/GFP + HDAd-SB and 12% for HDAd-mgmt/GFP + HDAd-SB. On day 3 after HDAd transduction, mice were sacrificed and GFP in BM LSK cells was analyzed. This reflects LSK cells that returned to the BM after mobilization and *in vivo* transduction. (Note that direct transduction of HSCs in the BM without mobilization is <0.5%.[4]) In the BM, ~2.5% and 3.5% GFP^+ LSK cells were found for HDAd-tHMGA2/GFP and HDAd-mgmt/GFP, respectively. The initial HSC transduction was not significantly different between the vectors for both time points. Based on this we concluded that GFP marking at week 44 was due to selective expansion of tHMGA2-expressing cells. Note that only a small fraction of GFP^+ cells will stably integrate the transgene cassette, because the *Sleeping Beauty* transposase system requires the co-infection of two vectors and Cre recombination overall is relatively inefficient.

In summary, *in vivo* HSC transduction with HDAd-tHMGA2 + HDAd-SB triggered a slow expansion at the HSC and progenitor levels.

GFP marking in secondary mice transplanted with Lin^- cells from HDAd-tHMGA2 + HDAdSB-transduced mice

To confirm that HDAd-tHMGA2/GFP-transduced primitive, long-term repopulating BM HSCs, we performed a transplantation/repopulation studyLin⁻ BM cells collected at week 44 after *in vivo* transduction of huCD46-transgenic mice were used for transplantation into lethally irradiated C57Bl/6 mice. The marking rate in Lin^- cells before transplantation was on average 15% (Figure 3A). Analysis of human CD46 expression on PBMCs at weeks 4 to 26 after transplantation showed engraftment rates of close to 100% (Figure S3A). Four weeks post transplantation, on average 40% of PBMCs expressed GFP and this fraction increased to ~65% by week 18 (Figure 3B). The percentage of GFP^+ MNCs in BM and spleen were on average 75% and 50% at week 18 in secondary mice (Figure 3C). For comparison, marking rates in *in vivo* transduced (primary) mice, where were ~43%, 10%, and 17% in PBMCs, BM-MNCs, and splenic MNCs, respectively, at week 44. On average 50% and 70% GFP expressing LSK cells were found in

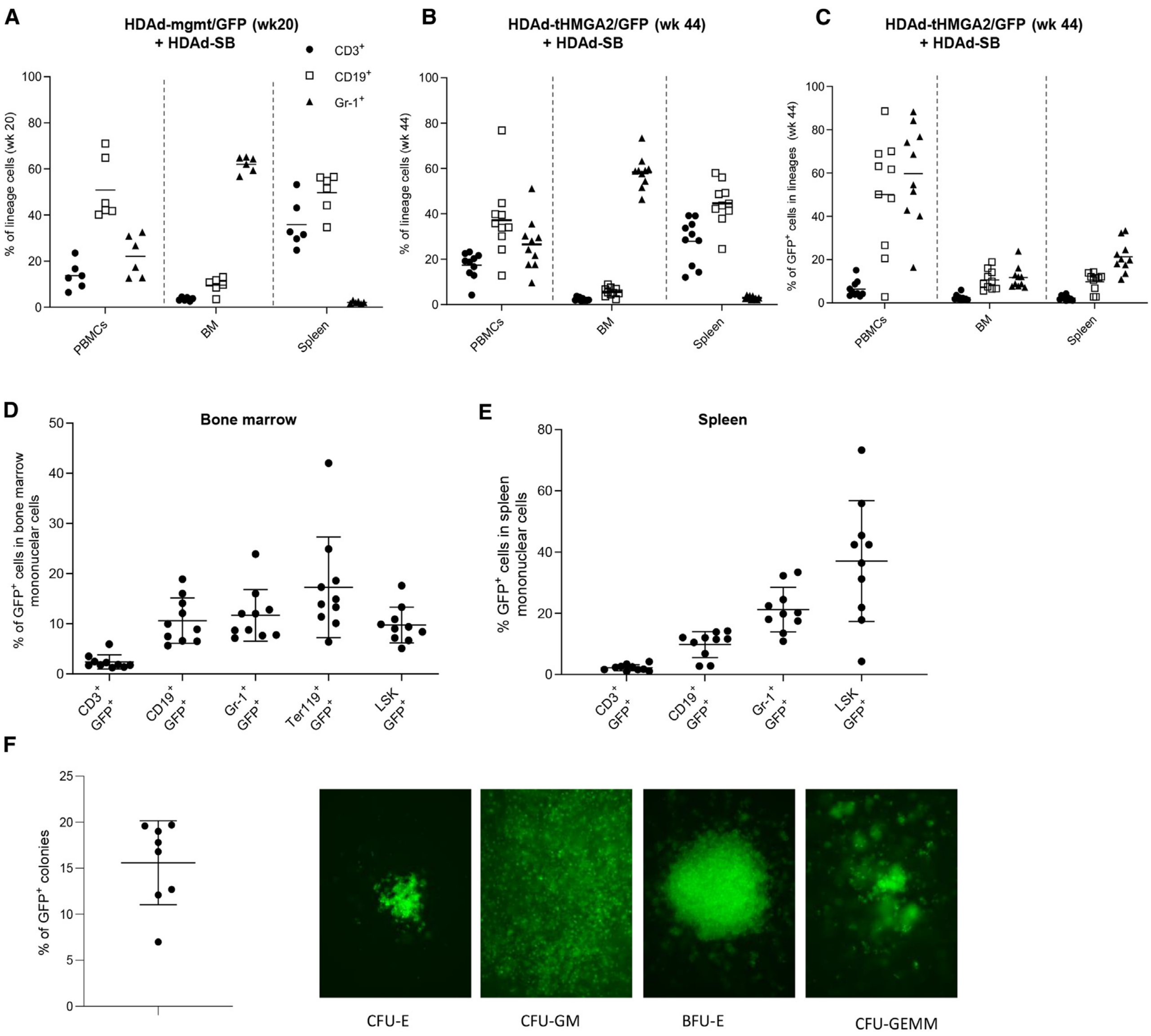

Figure 2. *In vivo* HSC transduction—analysis of bone marrow and spleen cells

(A) Percentage of lineage-positive cells within PBMCs, bone marrow, and spleen of the mice after *in vivo* transduction with HDAd-mgmt/GFP + HDAd-SB (sacrificed at week 20). (B) Percentage of lineage-positive cells within PBMCs, bone marrow, and spleen of the mice that were *in vivo* transduced with HDAd-tHMGA2/GFP + HDAd-SB (sacrificed at week 44). (C) Percentage of GFP^+ cells within lineage-positive cells in PBMCs, bone marrow, and spleen of the mice that were *in vivo* transduced with HDAd-tHMGA2/GFP + HDAd-SB (sacrificed at week 44). (D and E) Percentage of GFP^+ within lineage-positive cells and LSK cells (the HSC-containing $Lin^-Sca\text{-}1^+c\text{-}Kit^+$ fraction) in bone marrow and spleen of the mice that were *in vivo* transduced with HDAd-tHMGA2/GFP + HDAd-SB (week 44). Each symbol is an individual animal. Mean and error bars (±SEM) are shown. (F) Progenitor colony assay. A total of 1,250 plated Lin^- cells routinely formed ~250 individual colonies. Left panel: percentage of GFP^+ progenitor colonies that formed after plating bone marrow Lin^- cells from individual week 44 mice. Right panel: representative GFP^+ colonies. CFU-E, colony-forming unit-erythroid; BFU-E, burst-forming unit-erythroid; CFU-GM, colony-forming unit-granulocytes, macrophage; CFU-GEMM, colony-forming unit -granulocyte, erythroid, macrophage, megakaryocyte.

the BM and spleen of week 18 secondary mice (10% and 35% at week 44 in primary mice) (Figure 3D). Analysis of lineage-positive cells in secondary mice at week 18 showed nearly complete marking in $Gr1^+$ cells, 30%–40% marking in $CD3^+$ cells, and ~60% marking in $CD19^+$ cells in the periphery, BM, and spleen (Figures 3E and 3F). VCNs were comparable (~1.4 copies per cell) in PBMCs, spleen MNCs, and BM MNCs (Figure S3B).

To support the hypothesis that hematopoietic stem and progenitor cell (HSPC) expansion is driven by constitutive expression of

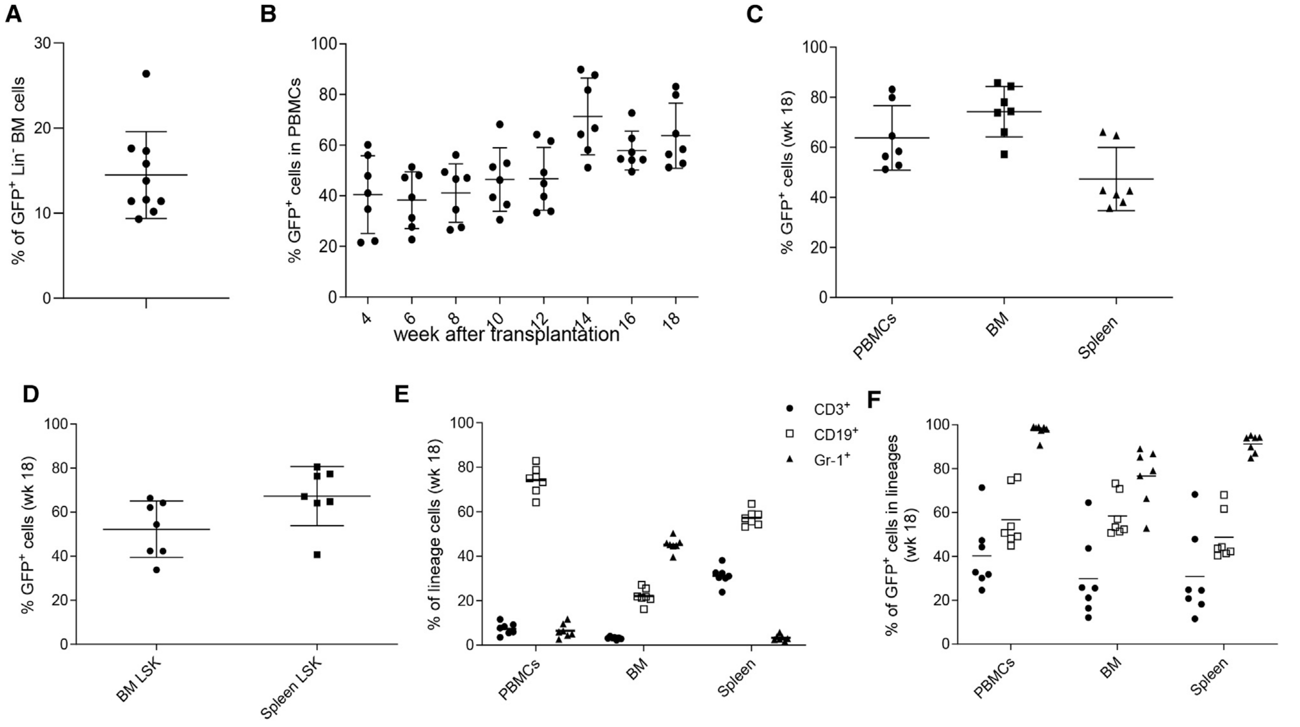

Figure 3. Analysis of secondary recipients transplanted with Lin⁻ cells from HDAd-tHMGA2+HDAdSB-transduced mice

(A) GFP marking rate in Lin⁻ bone marrow cells used for transplantation into secondary recipients. Each symbol represents an individual mouse. Mean and error bars (±SEM) are shown. (A) Percentage of GFP+ PBMCs. (B) Percentage of GFP+ cells in PBMCs in secondary recipients at different time points. (C) Percentage of GFP+ cells in total mononuclear cells of PBMC, bone marrow, and spleen at week 18 after transplantation. (D) Percentage of GFP+ cells within bone marrow LSK cells and spleen LSK cells. (E) Percentage of lineage-positive cells in PBMC, bone marrow, and spleen cells at week 18. (F) Percentage of GFP+, lineage-positive cells in PBMC, bone marrow, and spleen cells at week 18 after transplantation.

tHMGA2 we demonstrated the presence of the HMGA2 protein in PBMCs and BM MNCs of secondary recipients by western blot (Figure S3C).

Taking the data from PBMC, BM, and spleen MNC analyses in primary and secondary mice together, this indicates that HDAd-tHMGA2/GFP-transduced HSCs and progenitors further expanded after transplantation, underscoring their long-term repopulating capacity.

Notably, in our previous studies with HDAd5/35++-mgmt/GFP vectors, we did not observe expansion of GFP marking in secondary recipients.[14] This effect was found only in mice that were *in vivo* transduced with HDAd5/35++-tHMGA2/GFP.

Safety studies

Hematological and histological analyses were performed with samples from secondary recipients to assess potential detrimental effects of constitutive tHMGA2 expression. Blood cell counts (white blood cells, neutrophiles, lymphocytes, monocytes, eosinophiles, basophiles) at the end of the study (week 18 in secondary mice) were not significantly different from untransduced mice (Figure 4A, left panel). Analysis of erythroid parameters (RBCs, Hb, HCT, MCV, MCH, MCHC, and RDW) also did not show abnormalities (Figure 4, right panel). Blood smears did not indicate any blast formation or reticulocytosis (Figure 4B). Bone marrow smears were unremarkable (Figure 4B, right panel). Immunohistochemistry staining for GFP of spleen sections showed signals in ∼50% of cells, consistent with the flow data (Figure 4C). GFP+ cells were localized both in the red and white pulp. No leukemic lesions were visible. Overall, this indicates that tHMGA2-mediated HSPC expansion did not result in neoplastic events.

Integration site analysis

A more profound analysis of potential cellular transformation by tHMGA2 is assessment of clonal expansion based on unique SB100x-mediated vector integration sites. Genomic DNA (gDNA) from BM MNCs of week 44 primary mice with different rates of GFP marking in PBMCs (ranging from 25% to 65%) were subjected to genome-wide transgene integration site analysis using two methods: (1) inverse PCR to analyze integration junctions involves the digestion of gDNA with an endonuclease that cuts once within the vector and up- or downstream of the vector within chromosomal DNA. Fragments were re-ligated under conditions that promote intramolecular reaction. The ligation mixture was subjected to nested PCR using the using vector specific primers facing outwards

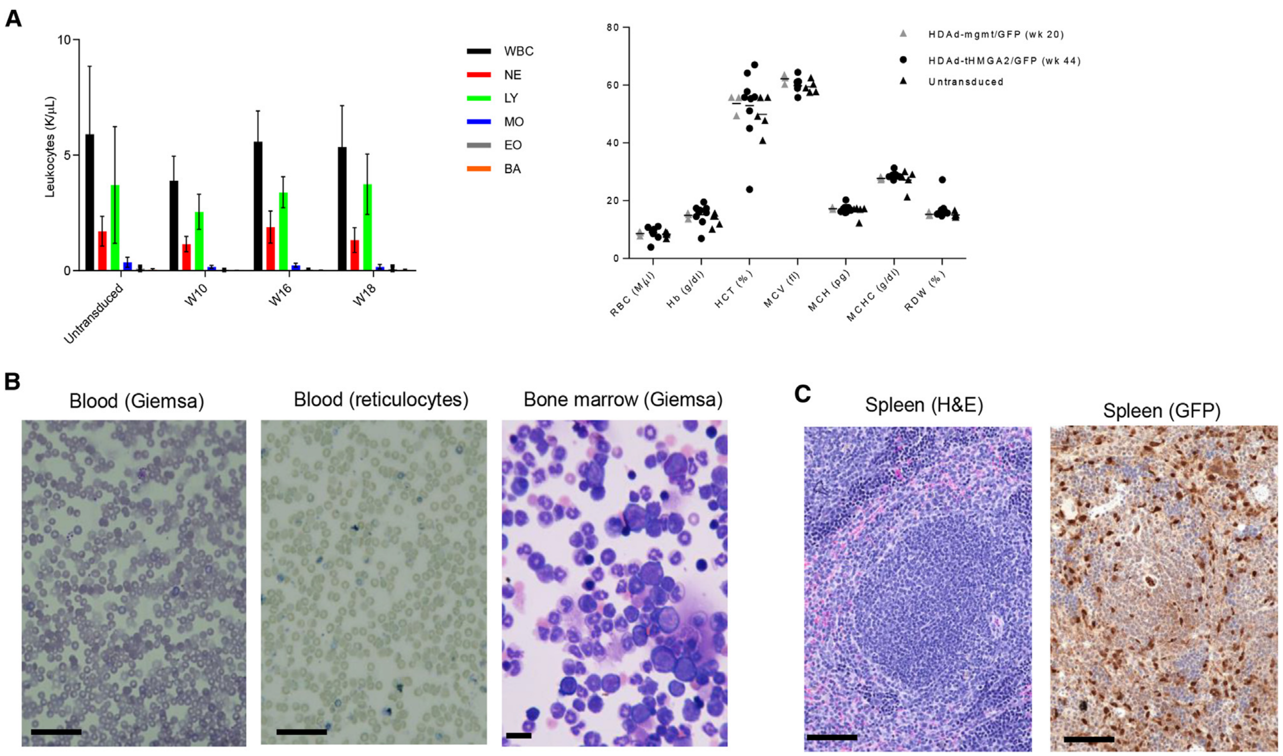

Figure 4. Hematological and histological assessment of neoplastic events in blood, bone marrow, and spleen of secondary recipients

(A) White blood cells of secondary mice at week 10, 16, and 18 after transplantation (left panel). Erythropoietic parameters of primary mice at sacrifice (right panel). RBC, red blood cells; Hb, hemoglobin; MCV, mean corpuscular volume; MCH, mean corpuscular hemoglobin; MCHC, mean corpuscular hemoglobin concentration; RDW, red cell distribution width. (B) Left panel: blood smears stained with Giemsa/May-Grünwald stain. Middle panel: blood smears stained with Brilliant cresyl blue for reticulocytes. Remnants of nuclei and cytoplasm in reticulocytes appear as purple staining. Right panel: bone marrow cytospins stained with Giemsa/May-Grünwald stain. (C) H&E staining of spleen sections. Scale bars, 20 μm.

(Figure S4A). PCR products showed a wide range of sizes with more than 10 distinct bands per DNA sample distinguishable (Figure S4B). Considering a VCN of 1.3 copies per cell, this indicates the presence of polyclonal expansion. (2) gDNA from BM MNCs were subjected to genome-wide transgene insertion site analysis to explore the number and clonal diversity of insertion events. For this we utilized the Illumina sequencing-based method transposase-assisted capture of transposable elements (TRACE),[28] which uses transposase tagmentation followed by two rounds of nested PCR to amplify fragments with junctions between the *Sleeping Beauty* transposase IR and adjacent DNA at sites of payload insertion. For GFP^+ samples 252–2,904 unique insertion site-containing DNA fragments were sequenced, which after alignment to the genome represented 33–158 unique genomic locations (Figure 5A). Sequence logos centered on these insertions reveal a palindromic motif consisting of a strong TA preference at the 0 and +1 positions, and a weaker preference for A and T in the −3 and +4 positions respectively (Figure 5B), consistent with the canonical *Sleeping Beauty* transposase motif.[29] Plotting the percentage of DNA fragments in support of each insertion site location shows high polyclonality and no dominant clones, with the most represented clone per sample only reaching 5.4%–20.6% of the population (Figure 5C). Analysis of insertion locations relative to gene features in treated mice shows that 26.6%–34.6% occurred in distal intergenic regions (Figure 5D). For insertion events proximal-to-or-within genes a majority were in introns (48.5%–60.3%) with lesser number present in promoter (6.03%–15.2%), exonic (0.94%–5.06%), 3′ UTR (0%–4.67%), or downstream (0%–3.03%) regions. In comparison, the mouse genome has a distribution of 44.8% distal intergenic, 39.7% intronic, 6.8% promoter, 4.9% downstream, 2.6% exonic, and 1.3% 3′ UTR (Figure 5D). A plot of chromosomes with insertion locations overlaid shows an even coverage of insertions among all chromosomes for a representative sample (Figure 5E); a pattern consistent for all GFP^+ samples (Figure S5). This near-random integration profile with a slight genic bias is consistent with previously published characterizations of SB and SB100x.[30–33] Taken together the above results suggest that tHMGA2-mediated enrichment results in polyclonal GFP^+ populations after random genomic insertion via *SB100x* transposase.

Analysis of genomic instability

Whole-exome sequencing was performed on BM DNA of mice stably expressing tHMGA2/GFP (week 18, secondary recipients) (N = 5) and untreated control mice (N = 3). Data were analyzed by GATK for variant calling and ANNOVAR for annotation.

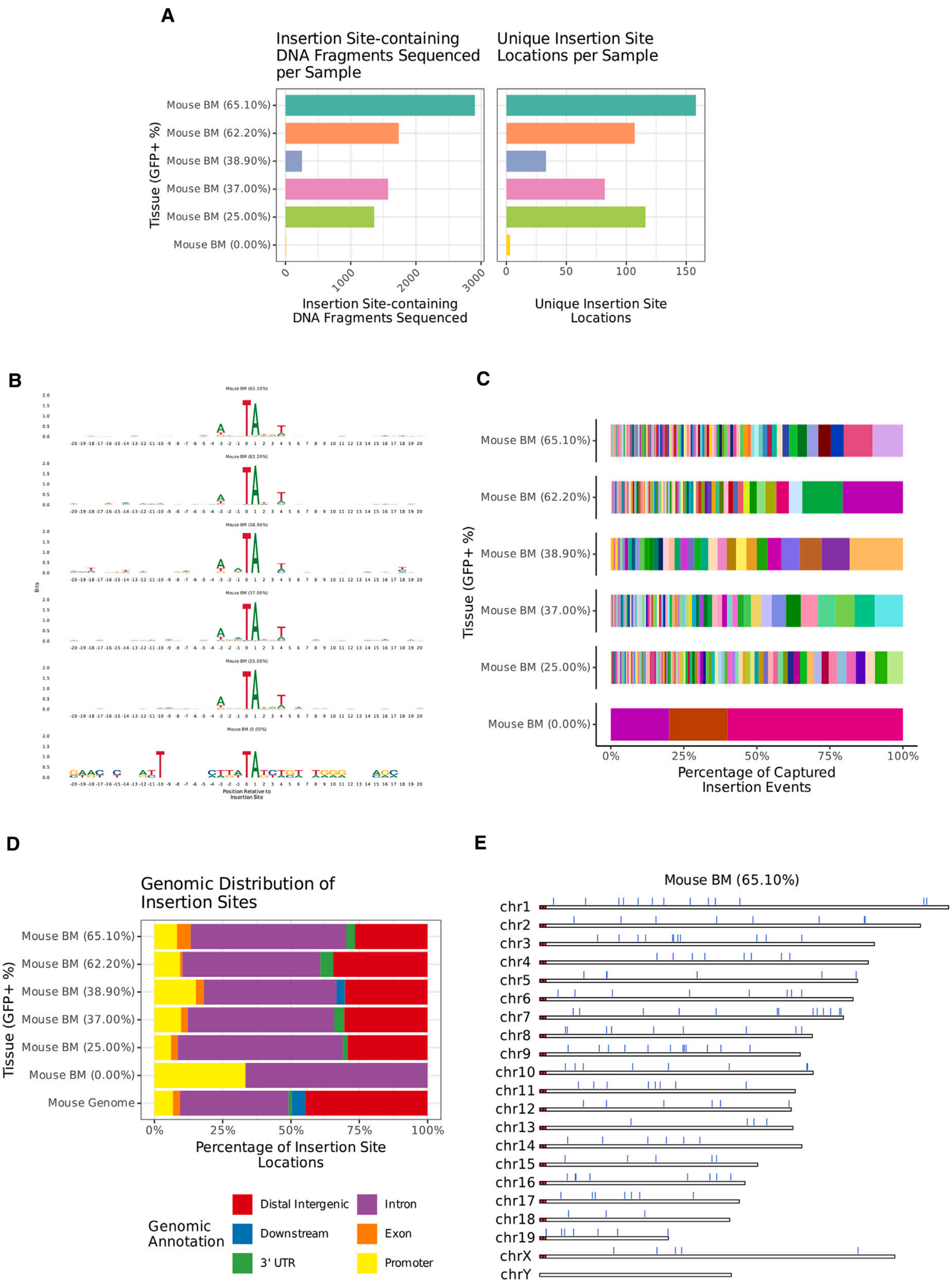

(legend on next page)

We classified the variants into two categories: (1) private variants, i.e., variants unique to each sample, meaning they are not found in any other treated or control samples, and (2) shared variants, i.e., variants that are found in at least one other treated or control sample. The Genomic Instability Index (GII) is proportion of private variants to the total number of variants. The GII between the two groups was not significantly different (Figures 6 and S6. This suggests that, in our study, continuous tHMGA2 expression and subsequent auto-expansion of HSPCs is not associated with a higher mutation risk.

In vivo transduction of human CD34$^+$ cells in humanized mice with HDAd-tHMGA2/GFP + HDAd-SB leads to *in vivo* expansion of the transduced cells

To assess whether tHMGA2 expression would also mediate *in vivo* expansion of human HSCS we performed *in vivo* transduction of human CD34$^+$ cells in humanized mice. In brief, our approach consists of transplantation of CD34$^+$ into partially myeloablated NOD.Cg-KitW-41J Tyr+ PrkdcscidIl2rgtm1Wjl/ThomJ (NBSGW) mice (12.5 mg/kg busulfan) to facilitate engraftment of human HSCs. Six weeks after transplantation, HSCs were mobilized with a 7-day mobilization scheme, including G-CSF 250 μg/kg i.p. (days 1–6) and AMD3100 5 mg/kg i.p. (days 5–7), as described previously,[34] followed by an i.v. injection of the HDAd-mgmt/GFP + HDAd-SB or HDAd-tHMGA2/GFP + HDAd-SB vector systems at the peak of mobilization. The mice were monitored for a period of 4 months post *in vivo* transduction, with bi-monthly assessment of GFP expression in the peripheral blood (Figure 7A). The 7-day mobilization scheme resulted in ~2 × 10^5 circulating CD34$^+$ cells in the periphery at the peak of mobilization (Figure 7B). *In vivo* HSC transduction of mobilized mice with integrating HDAd vectors resulted in low GFP marking in both HDAdmgmt/GFP and at week 12 that was comparable in both HDAd-mgmt/GFP + HDAdSB and HDAd-tHMGA2/GFP + HDAdSB groups (Figure 7C). However, at the end of the observation period (week 24), there were ~20% human GFP$^+$ PBMCs in HDAd-tHMGA2/GFP mice compared with ~2% in the HDAd-mgmt/GFP-transduced group. Four months post *in vivo* transduction, the mice were sacrificed, and the BM was harvested for analysis. At the time of sacrifice, multilineage engraftment in the BM was evident in all mice, with no discernible variations in BM lineages between the mgmt/GFP and tHMGA2/GFP mice (Figure 7D). Although not statically significant, the BM of tHMGA2/GFP-transduced mice contained more CD3$^+$ cells compared with the mgmt/GFP mice. That could be a result of the increased HSC proliferation and differentiation of HSCs because of the overexpression of tHMGA2, that could potentially lead to a greater pool of T cells within the BM (Figure 7D). Importantly, GFP marking in different lineages within the BM of tHMGA2/GFP mice exhibited superiority over their mgmt/GFP-transduced counterparts (Figure 7E). Notably, the percentage of GFP$^+$ CD34$^+$ cells was 26.94% ± 10.9% in tHMGA2/GFP-transduced mice vs. 5.25% ± 1.21% in mgmt/GFP-transduced mice (p = 0.09). Finally, the VCN within the hCD45$^+$ cells isolated from the chimeric BM was comparable between the mgmt/GFP and the tHMGA2/GFP mice, indicating that the increased GFP expression was attributable to *in vivo* expansion of transduced cells, rather than higher transduction efficiency of human HSCS by the HDAd-tHMGA2/GFP viral vector (Figure 7F).

DISCUSSION

We show that HSC mobilization and a single i.v. injection of an integrating HDAd vector system results in stable transduction of HSCs and expansion of HSCs and progenitors reaching average marking rates in PBMCs of ~50%. Notably, for hemoglobinopathies, 20% of corrected peripheral blood cells are sufficient for phenotypic correction.[25] This expansion was mediated by constitutive expression of tHMGA2 after random vector integration catalyzed by SB100x transposase. Expansion occurred at the level of HSCs as well as at the level of progenitors within the BM and, after their exit from these hematopoietic tissues, in the periphery. Expansion was seen in myeloid/granulocyte Gr-1$^+$ cells, B cell lymphoid CD19$^+$ cells, erythroid Ter119$^+$ cells and T cell lymphoid CD3$^+$ cells in *in vivo* transduced (primary) mice and secondary recipients. CD3$^+$ cell expansion was more pronounced in secondary than in primary animals. Importantly, expansion was polyclonal, based on integration site localization. Auto-expansion of tHMGA2 expressing cells seems to follow the reconstitution pattern after CD34$^+$ cell transplantation into myeloablated/conditioned recipients; with early recovery of granulocytes, NK cells, and B cells, and relative late expansion of T cells.[35]

Potential mechanisms for tHMGA2-mediated HSPCs *in vivo* expansion were previously discussed by Bonner et al.[22] They include (1) HMGA2 overexpression resulting in upregulation of insulin growth factor binding protein 2, a cell proliferation-stimulating protein with anti-apoptotic functions.[36,37] (2) HMGA2 stabilizing RNA replication forks in HSPC, thereby reducing apoptosis while also enhancing self-renewal.[38,39] It cannot be completely excluded that, in addition to tHMGA2-mediate effects on HSPCs, the treatment with immunosuppressive drugs leading to a change in the BM environment, has contributed to the HSPC proliferation in HDAd-tHMGA2-treated mice. It also remains to be investigated how

Figure 5. Genome-wide insertion site analysis by TRACE sequencing

(A) The number of unique DNA fragments sequenced with *Sleeping Beauty* IR-genome junctions, and the number of unique genomic coordinates where an insertion site was detected. Data from bone marrow MNCs of individual mice are shown. The percentage of GFP$^+$ bone marrow MNCs for each mouse is shown on the left side of the graphs. (B) Sequence logos ±20 bp from the insertion site, with zero being the first base downstream of the inserted transposon. (C) The percentage of unique insertion sites-containing DNA fragments mapping to a specific genomic coordinate. (D) Percentage of insertion locations contained within each gene region (relative to transcripts) including promoters (−2 to +0.2 kbp from transcript TSSs), exons, introns, 3′ UTRs, downstream (+3 kbp from 3′-most gene end), and distal intergenic (not contained within any of the previously mentioned features). Distribution of features over the entire mouse genome is included for comparison. (E) A representative plot of mouse chromosomes with tick marks denoting the location of insertion sites.

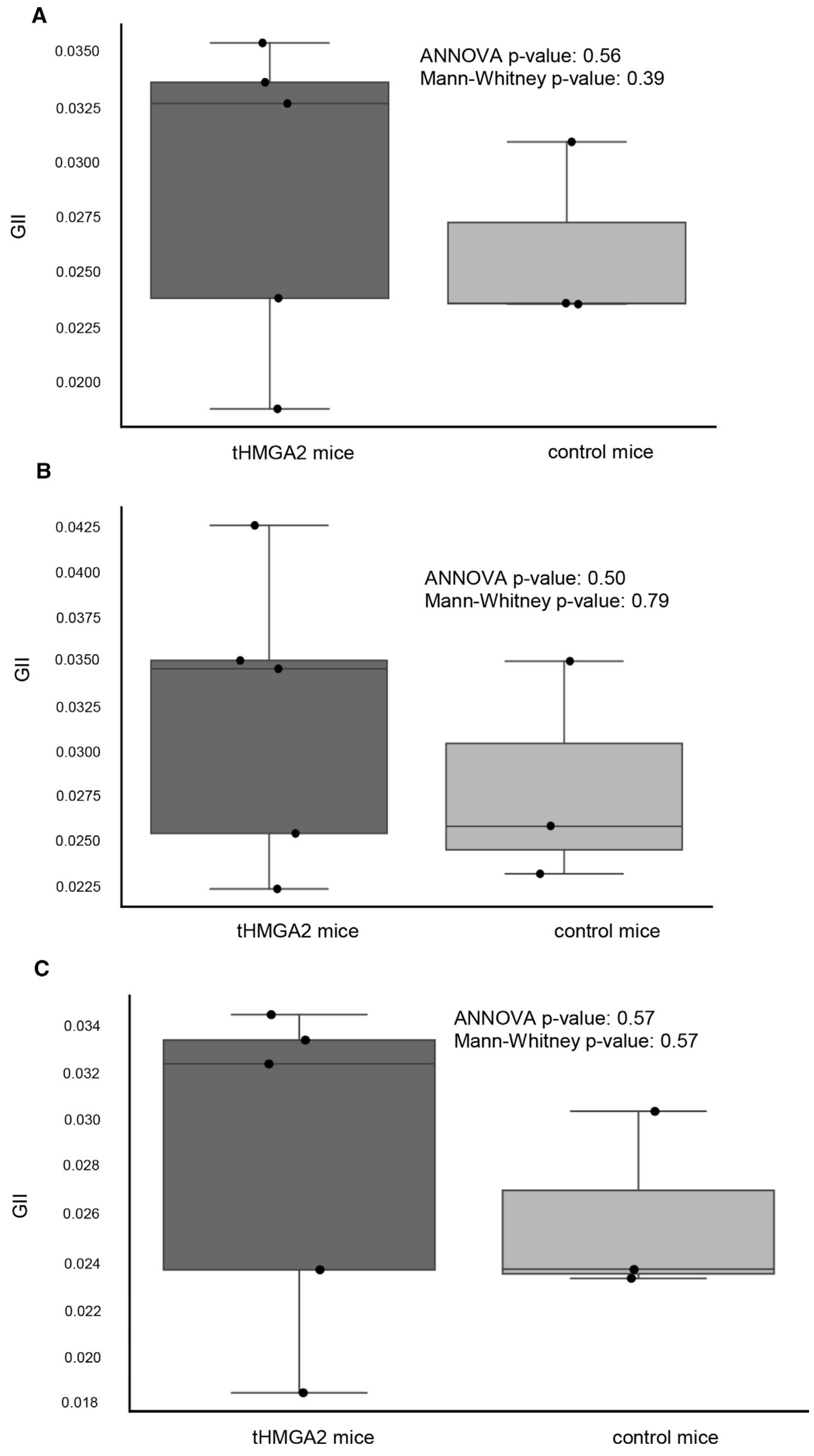

(legend on next page)

alterations in the BM niche occurring in Fanconi anemia and SCD will influence tHMGA2-triggered HSPC expansion.

On the other hand, HMGA2 overexpression is known for its association with epithelia-derived cancer[40] and hematological malignancies,[41–43] whereby it is likely that, in addition to HMGA2 overexpression, other mutations or changes in expression of driver genes are required for full tumorigenesis.[44] Furthermore, analysis of lentivirus vector integration sites in *ex vivo* HSPC gene therapy trials for SCID-X1 showed preferential integration within the intron 3 of the HMGA2 gene, which in one patient led to the expansion of HSPCs (and myeloid lineage) clones expressing truncated HMGA2, but without disturbance of hematopoiesis.[45] Wang et al. reported enrichment of HMGA2 clones in two SCID-X1 patients treated with γ retroviral gene therapy.[46] This could indicate that retroviral insertion-mediated activation of HMGA2 can provide a growth advantage for HSCs.

In agreement with studies in tHMGA2-transgenic mice and *ex vivo* HSC transduction studies using tHMGA2-expressing lentivirus vectors performed in NHPs,[21,22] we did not observe clonal expansion of HSPCs that constitutively overexpress tHMGA2. Notably, in contrast to lentivirus vectors, which preferentially integrate into active genes,[47] SB100x-mediated integration (used in our approach) is random and, therefore, theoretically safer. Furthermore, disconnecting the HMGA2 gene from its endogenous promoter might have contributed to the observed safety profile in mice.

Analysis of WES data from BM DNA samples of HDAd-tHMGA2-treated mice (week 18, secondary mice) and untreated control mice did not find significant differences in the genomic instability indices between the two groups, suggesting that the continuous tHMGA2 expression does not trigger premalignant settings. Further research with larger sample sizes and longitudinal analysis of *de novo* mutations is required to confirm this conclusion.

Another question that arises is whether increased proliferation of HSCs results in earlier exhaustion and thus increases the risk of BM failure. Additional more rigorous serial transplantation studies will be needed to address this question.

To our knowledge, there is no drug-free *in vivo* expansion system that could be used in the context of *ex vivo* and *in vivo* HSC gene therapy. Drug-resistance-based selection systems such on mgmt,[48] dhfr,[49] mdr1,[50] hprt,[51,52] or cytidine deaminase are associated with hematopoietic and/or extra-hematopoietic toxicity. Furthermore, recently made progress with epitope editing within CD123,[53] Flt3, CD123, cKit,[54] CD45[55] membrane receptors, in combination with antigen-specific, cell-depleting drug modalities, such as ADCs or chimeric antigen receptor, could come at the cost of unspecific toxicity and high treatment expenses. This raised interest in endogenous regulators of HSC proliferation and self-renewal such as HOXB4, Notch, and the Wnt/β-catenin pathways. Overexpression of HOXB4 has been shown to expand HSCs *in vivo* after *ex vivo* HSC transduction with lentivirus vectors but concerns regarding potential tumorigenicity stalled the development of this approach.[56] A potentially impactful approach is based on truncation in the erythropoietin receptor (tEPOR), in which the intracellular inhibitory domain to erythropoietin signaling is eliminated. This causes benign congenital erythrocytosis—a condition marked by a non-pathogenic hyper-production of red blood cells.[57,58] Uchida et al. reported that *ex vivo* tEPOR gene addition in HSCs or *ex vivo* editing to create a tEPOR can confer a selective advantage to the derived red blood cells, most likely due to mediating hypersensitivity to erythropoietin.[59]

Clearly, focusing on hematopoietic diseases in which correction of mutation or ectopic expression confers proliferative advantage, such as Fanconi anemia,[60,61] would be reasonable for *in vivo* HSC gene therapy because it would not require any drug- or transgene-mediated expansion.

Of note is also that the current G-CSF/AMD3100 mobilization regimen triggers leukocytosis and release of pro-inflammatory cytokines from granulocytes, which would be particularly critical in SCD patients.[62] Among the alternative G-CSF-free mobilization protocols that we have tested in the context of *in vivo* HSC transduction are tGroβ/AMD3100[63] and WU106/AMD3100.[64]

tHMGA2-mediated expansion was slow and did not reach the levels seen with O^6BG/BCNU-mediated expansion. In previous *in vivo* HSC transduction studies in CD46tg mice with HDAd-mgtm/GFP + HDAd.SB, after three rounds of O^6BG/BCNU given over an interval of 4–6 weeks, GFP marking in PBMCs reached 60%–90%.[14] We observed similar marking levels in peripheral blood cells with mgmt/γ-globin vectors.[18,65] Notably, in our 44-week study with HDAd-tHMGA2 + HDAd-SB, GFP marking in PBMCs did not reach a plateau.

In summary, we present an approach that mediated the auto-expansion of *in vivo* transduced HSPCs and myeloid, lymphoid, and erythroid progenitors without disturbance of hematopoiesis or neoplastic transformation. Like all the above listed technologies for *in vivo* expansion of gene-corrected HSPCs, our tHMGA2-based *in vivo* approach also requires long-term safety studies, optimally in non-human primates.

MATERIALS AND METHODS

Reagents for *in vivo* transduction and selection

G-CSF (Neupogen) (Amgen, Thousand Oaks, CA), AMD3100 (MilliporeSigma, Burlington, MA), and dexamethasone sodium

Figure 6. GII of mouse DNA

GII corresponds to the number of private variants compared with the total number of variants. Levels of GII were comparable between groups for (A) all variants ($p = 0.392$ by Mann-Whitney U test and $p = 0.563$ by ANOVA), (B) insertions and deletions (INDELs; $p = 0.786$ and $p = 0.497$), and (C) SNPs ($p = 0.571$ and $p = 0.575$).

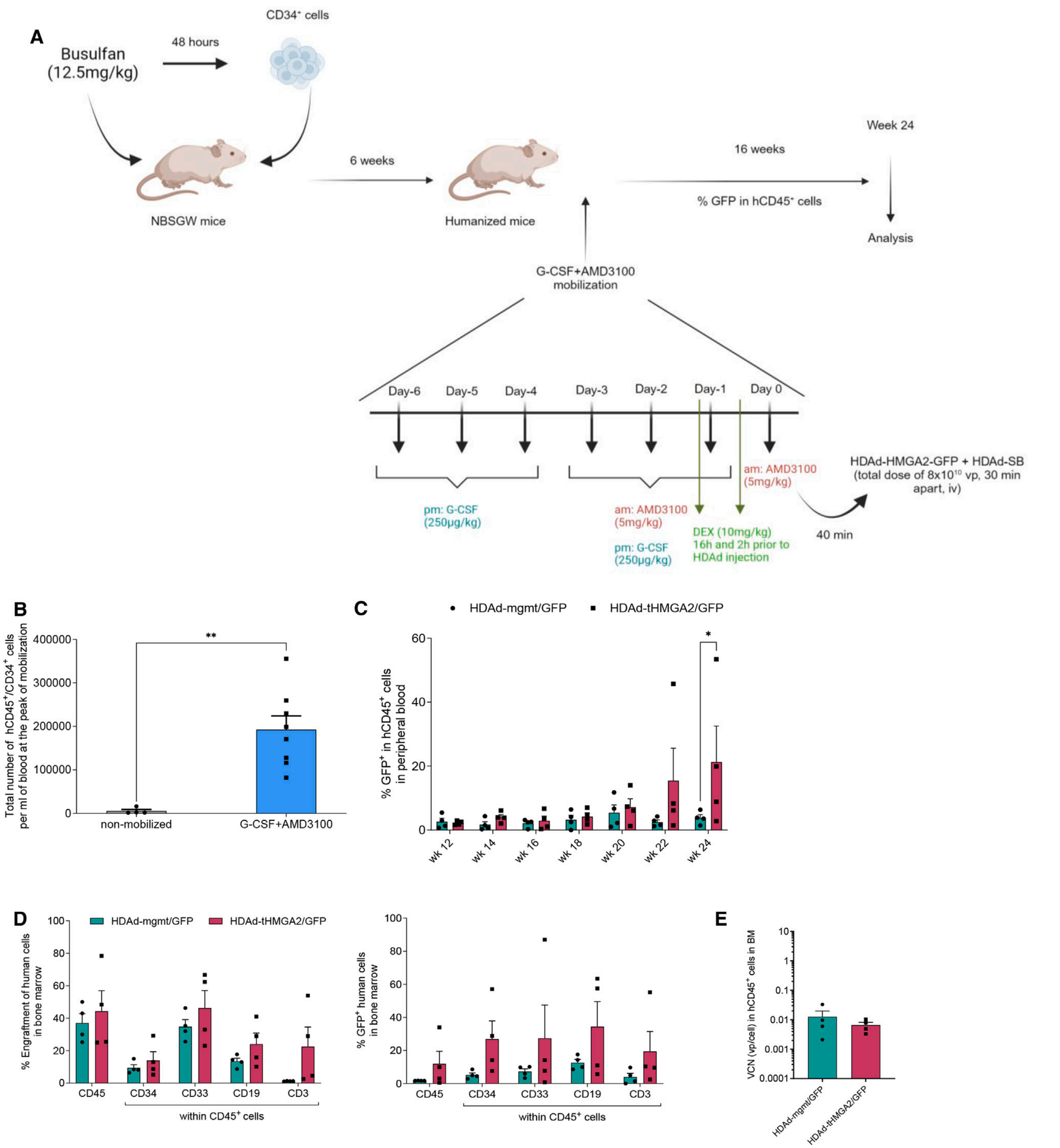

Figure 7. *In vivo* transduction of human CD34$^+$ cell by HDAd-tHMGA2/GFP in humanized mice

(A) Experimental procedure of *in vivo* transduction experiments. In brief, CD34$^+$ cells from healthy donors were transplanted in busulfan-treated NBSGW mice (*N* = 8). Six weeks post transplantation, CD34$^+$ cells were mobilized to the peripheral blood by G-CSF and AMD3100. At the peak of mobilization, the mice were injected i.v. with HDAd-mgmt/GFP + HDAd-SB (*N* = 4) or HDAd-tHMGA2/GFP + HDAd-SB (*N* = 4) adenoviral vectors, and were followed up for the next 4 months, at which time point the mice were euthanized, and their hematopoietic tissues were collected for further analysis. (B) Efficiency of G-CSF+AMD3100 mobilization in NBSGW mice xenotransplanted with human CD34$^+$ cells from healthy donors (*N* = 8), in terms of total hCD45$^+$/hCD34$^+$ cell number per mL of blood. (C) Percentage of GFP expression in human CD45$^+$ in the

(legend continued on next page)

phosphate (Fresenius Kabi USA, Lake Zurich, IL) were used. O^6-BG and BCNU were from Millipore/Sigma.

Construction of tHMGA2 vectors

To construct pHCA-PT4-tHMGA2/GFP vector, the $MGMT^{P140K}$ gene in the vector pHCA-PT4-mgmt/GFP[66] was replaced by the human HMGA2 gene (www.uniprot.org/uniprotkb/P52926/entry) by Gibson assembly (New England Biolabs). For the production of HDAd5/35++ vectors, corresponding plasmids were linearized with PmeI and rescued in 116 cells with AdNG163-5/35++, an Ad5/35++ helper vector containing chimeric fibers composed of the Ad5 fiber tail, the Ad35 fiber shaft, and the affinity-enhanced Ad35++ fiber knob. The HDAd-mgmt/GFP vector has been described previously.[14]

Animal studies

All experiments involving animals were conducted in accordance with the institutional guidelines set forth by the University of Washington. The studies were approved by the University of Washington IACUC (protocol no. 3108-01). C57BL/6-based transgenic mice that contained the human CD46 genomic locus and provide CD46 expression at a level and in a pattern similar to humans ($hCD46^{+/+}$ mice) were described earlier.[26] Long-term studies (44 weeks) are difficult to perform with males. Therefore, all mice were females.

HSC mobilization and* in vivo *transduction

HSCs were mobilized in mice by s.c. injections of human recombinant G-CSF (250 μg/kg/mouse/day, 4 days) followed by an s.c. injection of AMD3100 (5 mg/kg) on day 5. In addition, animals received dexamethasone (10 mg/kg, i.p.) 16 and 2 h before virus injection to blunt innate toxicity associated with i.v. HDAd injection. Forty-five minutes after AMD3100, animals were intravenously injected with virus vectors through the retro-orbital plexus (4×10^{10} viral particles per mouse).

Immunosuppression

Intraperitoneal injection of mycophenolate mofetil (20 mg/kg/day), rapamycin (0.2 mg/kg/day), and methylprednisolone (20 mg/kg/day) three times per week was performed.

Secondary BM transplantation

BM cells from *ex vivo* or *in vivo* transduced CD46tg mice were isolated aseptically. Lineage-depleted (Lin^-) cells were isolated and transplanted as described above. The secondary recipients were kept for 16 weeks after transplantation for terminal point analyses.

Mobilization and* in vivo *editing of $CD34^+$ cells in a humanized NBSGW mouse model

The immunodeficient NBSGW mice were obtained from The Jackson Laboratory (Bar Harbor, ME). A humanized model was generated by transplanting $CD34^+$ cells from healthy donors into partial myeloablated NBSGW mice (1×10^6/recipient) (busulfan, 12.5 mg/kg). Six weeks post transplantation, the mice, having a human BM chimerism, were mobilized by a 7-day mobilization scheme, including G-CSF 250 μg/kg i.p. (days 1–6) and AMD3100 5 mg/kg i.p. (days 5–7), as described previously.[32] Forty minutes post last AMD3100 injection, mice received an i.v. injection of the HDAd-mgmt/GFP or HDAd-tHMGA2/GFP vector along with HDAd-SB, at a total dose of 8×10^{10} viral particles (divided into two doses, 30 min apart). Sixteen and 2 h before i.v. injection of adenoviral vectors, the animals received dexamethasone (i.p., 10 mg/kg). The mice were followed up for 4 months post *in vivo* transduction, with bi-monthly assessment of GFP expression in the peripheral blood. Four months post *in vivo* transduction, NBSGW mice were sacrificed, and BM cells were collected, for assessment of multilineage engraftment and transgene expression.

Magnetic cell sorting

The human $CD45^+$ cells from chimeric BM, were isolated using human CD45 Microbeads (cat. no. 130-045-801) (Miltenyi Biotec, San Diego, CA) according to the manufacturer's instructions. The positive fractions were used for VCN analysis.

Flow cytometry

Cells were resuspended at 1×10^6 cells/100 μL in FACS buffer (PBS plus 1% heat-inactivated FBS) and incubated with FcR blocking reagent (Miltenyi Biotech, Auburn CA) for 10 min on ice. Next, the staining antibody solution was added in 100 μL per 10^6 cells and incubated on ice for 30 min in the dark. After incubation, cells were washed once in FACS buffer. For secondary staining, the staining step was repeated with a secondary staining solution. After the wash, cells were resuspended in FACS buffer and analyzed using an LSR II flow cytometer (BD Biosciences, San Jose, CA). Debris was excluded using a forward scatter-area and sideward scatter-area gate. Single cells were then gated using a forward scatter-height and forward scatter-width gate. Flow cytometry data were then analyzed using FlowJo (v.10.0.8, FlowJo). For analysis of LSK cells, cells were stained with biotin-conjugated lineage detection cocktail (cat. no. 130-092-613) (Miltenyi Biotec, San Diego, CA), antibodies against c-Kit (clone 2B8, cat. no. 12-1171-83) and Sca-1 (clone D7, cat. no. 25-5981-82), followed by secondary staining with APC-conjugated streptavidin (cat. no. 17-4317-82) (eBioscience, San Diego, CA). Other antibodies from eBioscience included anti-mouse CD3-APC (clone 17A2) (cat. no. 17-0032-82), anti-mouse CD19-PE-Cyanine7 (clone eBio1D3) (cat. no. 25-0193-82), and anti-mouse Ly-6G/Ly-6C (Gr-1)-PE (clone RB6-8C5) (cat. no. 12-5931-82). To evaluate the multilineage engraftment of $CD34^+$ cells in the BM of NBSGW mice post transplantation, the following antibodies were used: CD45-APC (BD Biosciences), CD19-PerCP (BioLegend), and

peripheral blood of HDAd-mgmt/GFP (or HDAd-tHMGA2/GFP *in vivo* transduced mice. (D) Left: multilineage reconstitution 4 months after *in vivo* transduction measured by flow cytometry with antibodies against human cell surface markers. Right: GFP expression of different human cell subpopulations from the chimeric bone marrow. (E) Vector copy number (VCN) analysis in isolated $hCD45^+$ cells from BM. Each symbol represents an individual mouse. Data are shown as means ± SEM. $^{**}p \leq 0.01$, $^{*}p \leq 0.05$ (two-way ANOVA with Bonferroni correction for multiple comparisons, Student's t test for two groups comparisons).

CD3-FITC (BioLegend), CD33-PE (BD Biosciences). To measure the percentage of human HSCs in peripheral blood of NBSGW mice post mobilization, the following antibodies were used: CD45-APC (BD Biosciences) and CD34-PE (BioLegend).

VCNs

Total DNA from PBMCs, BM, or spleen MNCs was extracted using the Quick-DNA miniprep kit (Zymo Research). Viral DNA extracted from purified HDAd virus stocks was serially diluted and used for a standard curve. qPCR was conducted in triplicate using the power SYBRTM Green PCR master mix on a StepOnePlus real-time PCR system (Applied Biosystems). A total of 9.6 ng DNA (9,600 pg/6 pg/cell = ∼1,600 cells) was used for a 10 μL reaction. The following primer pairs were used: mouse GAPDH forward, 5′-TTCCATCCT CCAGAAACCAG-3′, and reverse, 5′-GTTCTTCTCGGGCAAAAA TG-3'; GFP forward, 5′-TCGTGACCACCCTGACCTAC-3′, and reverse, 5′-GGTCTTGTAGTTGCCGTCGT-3'.

Western blot for tHMGA2

To show HMGA2 expression *in vivo*, PBMCs and BM cells from secondary mice collected at week 18 were used. Cells were lysed with Laemmli buffer (Bio-Rad) supplemented with fresh β-mercaptoethanol, subjected with sonication, separated by polyacrylamide gel electrophoresis, and then transferred onto nitrocellulose membranes. The blot was blocked in blocking buffer (TBS-3% milk) overnight, and then incubated with following antibodies: HMGA2 antibody (Cell Signaling), monoclonal anti-β-actin antibody (Millipore Sigma), or anti-GFP antibody (Cell Signaling). To visualize binding, the blot was incubated with anti-rabbit immunoglobin G (IgG) horseradish peroxidase (HRP) (Cell Signaling), or goat anti-mouse IgG HRP (BD Pharmingen) and developed with ECL Prime Western Blotting Detection Reagent (Amersham).

Integration site analysis: Inverse PCR

gDNA from week 44 BM MNCs was extracted using the Quick-DNA miniprep kit (Zymo Research). Integration junctions in gDNA were analyzed by inverse PCR as described elsewhere.[67] In brief, 5 μg gDNA was digested with EcoRI and religated under conditions that promote intramolecular reaction. The ligation mixture was subjected to phenol/chloroform extraction and precipitated with ethanol, resuspended in 50 μL TE buffer and then used in PCR as template. The following primers were used: P1a, 5-ctcactatagggcgaattggagctcAGTC TGTTTCACCTCGAGGTCTTCCTCAGC-3, and P1b, 5-CACTAA AGGGAACAAAAGCTGGTACCcgctcttcgagcagatatcataagatac-3. By using KOD Xtreme hot start DNA polymerase (Sigma-Aldrich), the following PCR program was used: 94°C for 2 min, 32 cycles of 98°C for 10 s and 60°C for 30 s, 68°C for 10 min.

Integration site analysis: TRACE

TRACE sequencing was performed as described previously[28] with the following modifications. Tagmentation was done in an 80 μL reaction by incubating 500 ng of gDNA with 3 μL of Tn5 transposase (Diagenode, C01070010) and 4.8 μL of annealed Tn5 adapter top and bottom oligos (Table S1) at 45 μM in 5X TAPs buffer (50 mM TAPS-NaOH [pH 8.5], 25 mM $MgCl_2$) with 8 μL DMF for 10 min at 55°C. The reaction was quenched with 160 μL RLT buffer (QIAGEN, 79216) followed by a 0.7X SPRI bead cleanup (Beckman Coulter, B23318). Tagmented DNA was eluted with 30 μL of nuclease-free water and 20 μL was used in the first round of PCR at 20 cycles with the Tn1c and LCI primers (Table S1) and Q5 High-Fidelity DNA Polymerase (NEB, M0494S), and the product was cleaned with 0.7X SPRI beads and eluted into 20 μL of nuclease-free water; 12 μL was then used for nested PCR at 20 cycles with primers MA-F and MA-LCII, and the product was purified with 0.7X SPRI-beads and eluted into 20 μL of nuclease-free water. Two microliters were used as input to indexing PCR with the NEBNext Multiplex Oligos for Illumina (Dual Index Primers Set 1) (NEB, E7600S) followed by a 0.7X SPRI bead cleanup. Final libraries were sequenced paired-end 150 × 2 (MiSeq Reagent Kit v.2 300-cycles; Ms-102-2002) on an Illumina MiSeq.

For computational analyses, unless otherwise stated default parameters are used for all commands. All code for the below initial TRACE processing is provided on GitHub (https://github.com/ensoma/andrelieber-trace-isa-wf). In brief, fastq trimming using cutadapt (https://doi.org/10.14806/ej.17.1.200) (v.4.4) was performed in four steps to ensure proper read structure: (1) R1 and R2 adapter trimming (-g “^NNNNCGAGTTTTAATGACTCCAACT” -G “^AGTGG CACAGCAGTTAGGNNNNNNNNAGATGTGTATAAGAGACAG” -m20 -e5), (2) IR trimming (-g “^TAAGTGTATGTAAACTTCC GACTTCAACTG” -m20 -e4), (3) R1 and R2 adapter readthrough trimming (-a “CTGTCTCTTATACACATCTNNNNNNNNCCTA ACTGCTGTGCCACT” -A “AGTTGGAGTCATTAAAACTCGNN NN” -m20 -e4), and (4) IR readthrough trimming (-A “CAGTTG AAGTCGGAAGTTTACATACACTTA” -m20 -e3). FastQC (https://www.bioinformatics.babraham.ac.uk/projects/fastqc/) (v.0.12.1) is then used for read QC before and after each processing step. The ENSEMBL mouse GRCm39 hard-masked assembly (https://ftp.ensembl.org/pub/release-111/fasta/musmusculus/dna/Musmusculus.GRCm39.dnarm.primaryassembly.fa.gz) and v.111 annotation (https://ftp.ensembl.org/pub/release-111/gtf/musmusculus/Musmusculus.GRCm39.111.chr.gtf.gz) were concatenated to the payload sequence, indexed with bwa (https://doi.org/10.48550/arXiv.1303.3997) (0.7.17), and the previously trimmed and filtered reads aligned to the index with bwa-mem. There are three samtools[68] (v.1.17) alignment filtering steps to ensure accurate insertion sites: (1) flag and quality score filtering (samtools view –q30 –F 3852 –f3; samtools sort –n; samtools fixmate –m; samtools view –f2; samtools sort), (2) removal of PCR duplicates (samtools sort –n; samtools fixmate –m; samtools sort; samtools markdup –r), (3) removing read-pairs with more than 5 soflcip reads on the 5′ end of R1 (a custom python [v.3.10.13] and pysam [https://github.com/pysam-developers/pysam] [v.0.21.0] script; samtools sort –n; samtools fixmate; samtools view –f3; samtools sort). Samtools flagstat and idstat are used before and after each filtering step to collect various metrics about the alignments. Insertion sites are called using a custom Python and Pysam script that finds the genomic coordinate of the 5′-most non-sofclipped base on the R1 read. Insertion sites within 5 bases are summed and the new location

set at the position with the largest read number or given equal read numbers for multiple positions of the median position of those sites. Insertion sites with a score ≥ 2 in two or more samples are removed using bedtools[69] (v.2.31.0) due to their likely status of being artifacts of the TRACE method. Insertion sites are annotated to the nearest gene using a custom R (v.4.3.1) script that utilizes ChIPseeker[70] (v.1.36.0) with the settings: tssRegion = c(–2000, 200), level = “transcript”.

R (v.4.3.2) was used for all downstream data analysis, and the code is provided on GitHub (https://github.com/ensoma/andrelieber-trace-isa-analysis). Insertion sites were retained if present in at least two of three technical replicates. ggplot2 (v.3.4.4) was used to plot the number of unique insertion site locations, number of captured insertion events, percentage of insertion events per insertion site, and distribution of insertion sites relative to genomic features. The percentage of the mouse genome contained within genomic features was calculated using a custom R script using GenomicFeatures (v.1.54.1),[71] GenomicRanges (v.1.54.1),[71] and Rsamtools (v.2.18.0) (https://doi.org/10.18129/B9.bioc.Rsamtools). Sequence logos were generated with ggseqlogo[72] (v.0.2) after sequences surrounding insertion sites were extracted from the fasta assembly using bedtools slop –b20 and bedtools getfasta –s. KaryoploteR[73] (v.1.28.0) genome = “mm39” was used to create the karyotype plots. Genome-wide insertion sites are shown in Table S2.

Whole-exome sequencing

Whole-exome sequencing (WES) was performed by CD Genomics on BM DNA samples from five mice treated with HDAd-tHMGA2+HDAd-SB (week 18 secondary mice) (BM1-BM5) and three untreated control mice (N1-N3). The raw sequencing data were filtered through a series of filtration methods to obtain high quality data. The statistics of the coverage on target exon regions are shown in Table S3.

WES data analysis for genomic instability

Bowtie2 was used to align the sequencing data to the reference genome mm39. Subsequently, variant calling was done by GATK (Genome Analysis Toolkit),[74] and the variants were annotated using ANNOVAR.[75] Variants were classified into two categories: private variants, which are unique to one sample, and shared variants, which appear in multiple samples. Then, single-nucleotide polymorphisms (SNPs) and insertions and deletions (INDELs), were distinguished by Bcftools. The GII was calculated for each sample using the formula: GII = number of private variants divided by the total number of variants. ANOVA and Mann-Whitney U tests were used to compare the GII between the HDAd-tHMGA2 and control groups for all variants, SNPs, and INDELs. In addition to p values, F statistic, and U statistic were calculated. The F statistic measures the ratio of variances within the groups. A higher F statistic means that higher variances occur when the individual data points tend to fall further from the mean. The U test values provide a measure of the rank-sum difference between the two groups and indicate that the observed differences are likely due to random chance, rather than a true effect of the treatment.

Blood analysis

Blood samples were collected into EDTA-coated tubes and analysis was performed on a HemaVet 950FS (Drew Scientific, Waterbury, CT). Peripheral blood smears were stained with Giemsa/May-Grünwald (Merck, Darmstadt, Germany) for 5 and 15 min, respectively. Reticulocytes were stained with Brilliant cresyl blue.

Cytospin slide preparation

Cytospins of 0.3–1.0 $\times$ 10^5 cells were prepared by cytocentrifugation (ROTOFIX 32, Hettich Zentrifugen) at 500 rpm for 5 min. Cytospins were air dried and then stained with Giemsa/May-Grünwald (Merck, Darmstadt, Germany) for 3–8 min, and subjected to imaging analysis.

Colony-forming unit assay

Lineage minus (Lin^-) cells were isolated by depletion of lineage-committed cells in BM MNCs using the mouse lineage cell depletion kit (Miltenyi Biotec) according to the manufacturer’s instructions. Colony-forming unit (CFU) assays were performed using ColonyGEL 1202 (Reachbio, Seattle, WA) with mouse complete medium according to the manufacturer’s protocol. Colonies were scored 10 days after plating.

Tissue analysis

Spleen tissue sections of 2.5 μm thickness were fixed in 4% formaldehyde for at least 24 h, dehydrated, and embedded in paraffin. Staining with hematoxylin and eosin was used for histological evaluation of neoplastic hemopoiesis. For GFP immunohistochemistry, paraffin sections were deparaffinized and hydrated through immersion in xylene, decreasing concentrations of ethanol (100%–95%–80%––70%), and water. Slides were then immersed in 0.3% hydrogen peroxide, followed by an additional rinse of water to eliminate endogenous peroxidase. Slides were then placed in 1% Unmasking solution (Vector Labs) and placed in a miniature autoclave (at up to 125°C for 1 h) for antigen retrieval. Slides were incubated in 2.5% normal horse serum (NHS) blocking solution (Vector Labs) for 20 min at room temperature, followed by incubation with the primary anti-body, rabbit anti-GFP (Cell Signaling, no. 2956S) (diluted 1:100 in PBS/1% NHS) overnight at 4°C. An ImmPress-AP Horse anti-Rabbit IgG polymer detection kit (no. MP-5401) was used. Following an additional wash in PBS, four drops of ImmPRESS Reagent Kit anti-goat Ig (Vector Labs) were added, and slides were incubated for 30 min. After washing, two drops of Polink-2 HRP Kit with DAB Chromogen (Golden Bridge International) were added and allowed to develop for around 5 min before washing with water. Sections were counterstained with Mayer’s hematoxylin (Sigma-Aldrich, St. Louis, MO) for 5 to 10 s and washed with water. After the slides dried, three to five drops of VectaMount (Vector Labs) were added to the slide with a coverslip placed on top. Images were taken with a Leica DMLB microscope (Wetzlar), using Leica DFC300 FX Digital camera and Leica Application Suite v.2.4.1 R1 software.

Statistical analyses

Statistical significance was calculated by appropriate statistical tests as described in the figure legends. Statistical analysis was computed on

GraphPad Prism v.9.0.0 (GraphPad Software, La Jolla, CA). $p < 0.05$ was considered as statistically different.

DATA AND CODE AVAILABILITY

All raw and processed sequencing data have been deposited into the NCBI gene expression omnibus (GEO) and sequencing read archive (SRA) under the accession number GSE268678.

The SRA accession number for the whole-exome sequences is PRJNA1136495. In addition, all code used to process and analyze the sequencing results is available on GitHub in the provided links.

ACKNOWLEDGMENTS

The study was supported by NIH grants R01 HL130040 and R01 AI17430 (to A.L.), by a grant from Ensoma Inc. (to A.L.), and by a grant from the Bill and Melinda Gates Foundation: INV-017692 (to A.L.). Under the grant conditions of the BMGF, a Creative Commons Attribution 4.0 Generic License has already been assigned to the Author Accepted Manuscript version that might arise from this submission. We thank Sucheol Gil and Theo Koob for technical support. We are grateful to Sarah Funk for providing NSGBW mice.

AUTHOR CONTRIBUTIONS

A.L. provided the conceptual framework for the study. H.W., A.G., R.P., and A.L. designed the experiments. H.W., A.G., K.W.M., and R.E. performed the experiments. E.N. and P.N.V. performed the WES data analysis. R.P. analyzed the insertion site data and provided critical comments on the manuscript. A.L. wrote the manuscript.

DECLARATION OF INTERESTS

A.L. and P.N.V. receive research funding from Ensoma, Inc. R.E., K.W.M., and R.P. are employees of Ensoma, Inc.

SUPPLEMENTAL INFORMATION

Supplemental information can be found online at https://doi.org/10.1016/j.omtm.2024.101319.

REFERENCES

1. Wang, H., Liu, Y., Li, Z., Tuve, S., Stone, D., Kalyushniy, O., Shayakhmetov, D., Verlinde, C.L.M., Stehle, T., McVey, J., et al. (2008). In vitro and *in vivo* properties of adenovirus vectors with increased affinity to CD46. J. Virol. *82*, 10567–10579. https://doi.org/10.1128/JVI.01308-08.

2. Yao, J., Atasheva, S., Wagner, N., Di Paolo, N.C., Stewart, P.L., and Shayakhmetov, D.M. (2024). Targeted, safe, and efficient gene delivery to human hematopoietic stem and progenitor cells *in vivo* using the engineered AVID adenovirus vector platform. Mol. Ther. *32*, 103–123. https://doi.org/10.1016/j.ymthe.2023.10.023.

3. Ni, S., Gaggar, A., Di Paolo, N., Li, Z.Y., Liu, Y., Strauss, R., Sova, P., Morihara, J., Feng, Q., Kiviat, N., et al. (2006). Evaluation of adenovirus vectors containing serotype 35 fibers for tumor targeting. Cancer Gene Ther. *13*, 1072–1081.

4. Richter, M., Saydaminova, K., Yumul, R., Krishnan, R., Liu, J., Nagy, E.E., Singh, M., Izsvák, Z., Cattaneo, R., Uckert, W., et al. (2016). In vivo transduction of primitive mobilized hematopoietic stem cells after intravenous injection of integrating adenovirus vectors. Blood *128*, 2206–2217. https://doi.org/10.1182/blood-2016-04-711580.

5. Li, C., Mishra, A.S., Gil, S., Wang, M., Georgakopoulou, A., Papayannopoulou, T., Hawkins, R.D., and Lieber, A. (2019). Targeted Integration and High-Level Transgene Expression in AAVS1 Transgenic Mice after In Vivo HSC Transduction with HDAd5/35++ Vectors. Mol. Ther. *27*, 2195–2212. https://doi.org/10.1016/j.ymthe.2019.08.006.

6. van Rensburg, R., Beyer, I., Yao, X.Y., Wang, H., Denisenko, O., Li, Z.Y., Russell, D.W., Miller, D.G., Gregory, P., Holmes, M., et al. (2013). Chromatin structure of two genomic sites for targeted transgene integration in induced pluripotent stem cells and hematopoietic stem cells. Gene Ther. *20*, 201–214. https://doi.org/10.1038/gt.2012.25.

7. Lazzarotto, C.R., Malinin, N.L., Li, Y., Zhang, R., Yang, Y., Lee, G., Cowley, E., He, Y., Lan, X., Jividen, K., et al. (2020). CHANGE-seq reveals genetic and epigenetic effects on CRISPR-Cas9 genome-wide activity. Nat. Biotechnol. *38*, 1317–1327. https://doi.org/10.1038/s41587-020-0555-7.

8. Liu, G., Yin, K., Zhang, Q., Gao, C., and Qiu, J.L. (2019). Modulating chromatin accessibility by transactivation and targeting proximal dsgRNAs enhances Cas9 editing efficiency *in vivo*. Genome Biol. *20*, 145. https://doi.org/10.1186/s13059-019-1762-8.

9. Li, C., Georgakopoulou, A., Newby, G.A., Everette, K.A., Nizamis, E., Paschoudi, K., Vlachaki, E., Gil, S., Anderson, A.K., Koob, T., et al. (2022). In vivo base editing by a single i.v. vector injection for treatment of hemoglobinopathies. JCI Insight *7*, e162939. https://doi.org/10.1172/jci.insight.162939.

10. Li, C., Georgakopoulou, A., Newby, G.A., Chen, P.J., Everette, K.A., Paschoudi, K., Vlachaki, E., Gil, S., Anderson, A.K., Koob, T., et al. (2023). In vivo HSC prime editing rescues sickle cell disease in a mouse model. Blood *141*, 2085–2099. https://doi.org/10.1182/blood.2022018252.

11. Haddad, E., Landais, P., Friedrich, W., Gerritsen, B., Cavazzana-Calvo, M., Morgan, G., Bertrand, Y., Fasth, A., Porta, F., Cant, A., et al. (1998). Long-term immune reconstitution and outcome after HLA-nonidentical T-cell-depleted bone marrow transplantation for severe combined immunodeficiency: a European retrospective study of 116 patients. Blood *91*, 3646–3653.

12. Fitzhugh, C.D., Cordes, S., Taylor, T., Coles, W., Roskom, K., Link, M., Hsieh, M.M., and Tisdale, J.F. (2017). At least 20% donor myeloid chimerism is necessary to reverse the sickle phenotype after allogeneic HSCT. Blood *130*, 1946–1948. https://doi.org/10.1182/blood-2017-03-772392.

13. Crone, T.M., Goodtzova, K., Edara, S., and Pegg, A.E. (1994). Mutations in human O6-alkylguanine-DNA alkyltransferase imparting resistance to O6-benzylguanine. Cancer Res. *54*, 6221–6227.

14. Wang, H., Richter, M., Psatha, N., Li, C., Kim, J., Liu, J., Ehrhardt, A., Nilsson, S.K., Cao, B., Palmer, D., et al. (2018). A Combined In Vivo HSC Transduction/Selection Approach Results in Efficient and Stable Gene Expression in Peripheral Blood Cells in Mice. Mol. Ther. Methods Clin. Dev. *8*, 52–64. https://doi.org/10.1016/j.omtm.2017.11.004.

15. Wang, H., Li, C., Obadan, A.O., Frizzell, H., Hsiang, T.-Y., Gil, S., Germond, A., Fountain, C., Baldessari, A., Roffler, S., et al. (2022). In vivo HSC gene therapy for SARS-CoV2 infection using a decoy receptor. Hum. Gene Ther. *33*, 389–403.

16. Li, C., Wang, H., Gil, S., Germond, A., Fountain, C., Baldessari, A., Kim, J., Liu, Z., Georgakopoulou, A., Radtke, S., et al. (2022). Safe and efficient *in vivo* hematopoietic stem cell transduction in nonhuman primates using HDAd5/35++ vectors. Mol. Ther. Methods Clin. Dev. *24*, 127–141. https://doi.org/10.1016/j.omtm.2021.12.003.

17. Li, C., Anderson, A.K., Wang, H., Gil, S., Kim, J., Huang, L., Germond, A., Baldessari, A., Nelson, V., Bar, K.J., et al. (2023). Stable HIV decoy receptor expression after *in vivo* HSC transduction in mice and NHPs: Safety and efficacy in protection from SHIV. Mol. Ther. *31*, 1188. https://doi.org/10.1016/j.ymthe.2023.02.019.

18. Wang, H., Georgakopoulou, A., Li, C., Liu, Z., Gil, S., Bashyam, A., Yannaki, E., Anagnostopoulos, A., Pande, A., Izsvák, Z., et al. (2020). Curative *in vivo* hematopoietic stem cell gene therapy of murine thalassemia using large regulatory elements. JCI Insight *5*, e139538. https://doi.org/10.1172/jci.insight.139538.

19. Fusco, A., and Fedele, M. (2007). Roles of HMGA proteins in cancer. Nat. Rev. Cancer *7*, 899–910. https://doi.org/10.1038/nrc2271.

20. Kumar, P., Beck, D., Galeev, R., Thoms, J.A.I., Talkhoncheh, M.S., de Jong, I., Unnikrishnan, A., Baudet, A., Subramaniam, A., Pimanda, J.E., and Larsson, J. (2019). HMGA2 promotes long-term engraftment and myeloerythroid differentiation of human hematopoietic stem and progenitor cells. Blood Adv. *3*, 681–691. https://doi.org/10.1182/bloodadvances.2018023986.

21. Ikeda, K., Mason, P.J., and Bessler, M. (2011). 3’UTR-truncated Hmga2 cDNA causes MPN-like hematopoiesis by conferring a clonal growth advantage at the level of HSC in mice. Blood *117*, 5860–5869. https://doi.org/10.1182/blood-2011-02-334425.

22. Bonner, M.A., Morales-Hernández, A., Zhou, S., Ma, Z., Condori, J., Wang, Y.D., Fatima, S., Palmer, L.E., Janke, L.J., Fowler, S., et al. (2021). 3’ UTR-truncated HMGA2 overexpression induces non-malignant *in vivo* expansion of hematopoietic stem cells in non-human primates. Mol. Ther. Methods Clin. Dev. *21*, 693–701. https://doi.org/10.1016/j.omtm.2021.04.013.

23. Li, C., Psatha, N., Wang, H., Singh, M., Samal, H.B., Zhang, W., Ehrhardt, A., Izsvák, Z., Papayannopoulou, T., and Lieber, A. (2018). Integrating HDAd5/35++ vectors as a new platform of HSC gene therapy of hemoglobinopathies. Mol. Ther. Methods Clin. Dev. *9*, 142–152. https://doi.org/10.1016/j.omtm.2018.02.004.

24. Li, C., and Lieber, A. (2019). Adenovirus vectors in hematopoietic stem cell genome editing. FEBS Lett. *593*, 3623–3648. https://doi.org/10.1002/1873-3468.13668.

25. Ni, S., Bernt, K., Gaggar, A., Li, Z.Y., Kiem, H.P., and Lieber, A. (2005). Evaluation of biodistribution and safety of adenovirus vectors containing group B fibers after intravenous injection into baboons. Hum. Gene Ther. *16*, 664–677.

26. Kemper, C., Leung, M., Stephensen, C.B., Pinkert, C.A., Liszewski, M.K., Cattaneo, R., and Atkinson, J.P. (2001). Membrane cofactor protein (MCP; CD46) expression in transgenic mice. Clin. Exp. Immunol. *124*, 180–189. https://doi.org/10.1046/j.1365-2249.2001.01458.x.

27. Boehme, P., Zhang, W., Solanki, M., Ehrke-Schulz, E., and Ehrhardt, A. (2016). A High-Capacity Adenoviral Hybrid Vector System Utilizing the Hyperactive Sleeping Beauty Transposase SB100X for Enhanced Integration. Mol. Ther. Nucleic Acids *5*, e337. https://doi.org/10.1038/mtna.2016.44.

28. Patel, S.H., Christodoulou, C., Weinreb, C., Yu, Q., da Rocha, E.L., Pepe-Mooney, B.J., Bowling, S., Li, L., Osorio, F.G., Daley, G.Q., and Camargo, F.D. (2022). Lifelong multilineage contribution by embryonic-born blood progenitors. Nature *606*, 747–753. https://doi.org/10.1038/s41586-022-04804-z.

29. Ivics, Z., and Izsvak, Z. (2015). Sleeping Beauty Transposition. Microbiol. Spectr. *3*, MDNA3-0042-2014. https://doi.org/10.1128/microbiolspec.MDNA3-0042-2014.

30. Yant, S.R., Wu, X., Huang, Y., Garrison, B., Burgess, S.M., and Kay, M.A. (2005). High-resolution genome-wide mapping of transposon integration in mammals. Mol. Cell Biol. *25*, 2085–2094. https://doi.org/10.1128/MCB.25.6.2085-2094.2005.

31. Moldt, B., Miskey, C., Staunstrup, N.H., Gogol-Döring, A., Bak, R.O., Sharma, N., Mátés, L., Izsvák, Z., Chen, W., Ivics, Z., and Mikkelsen, J.G. (2011). Comparative genomic integration profiling of Sleeping Beauty transposons mobilized with high efficacy from integrase-defective lentiviral vectors in primary human cells. Mol. Ther. *19*, 1499–1510. https://doi.org/10.1038/mt.2011.47.

32. Ye, L., Lam, S.Z., Yang, L., Suzuki, K., Zou, Y., Lin, Q., Zhang, Y., Clark, P., Peng, L., and Chen, S. (2024). AAV-mediated delivery of a Sleeping Beauty transposon and an mRNA-encoded transposase for the engineering of therapeutic immune cells. Nat. Biomed. Eng. *8*, 132–148. https://doi.org/10.1038/s41551-023-01058-6.

33. Miskey, C., Kesselring, L., Querques, I., Abrusán, G., Barabas, O., and Ivics, Z. (2022). Engineered Sleeping Beauty transposase redirects transposon integration away from genes. Nucleic Acids Res. *50*, 2807–2825. https://doi.org/10.1093/nar/gkac092.

34. Psatha, N., Sgouramali, E., Gkountis, A., Siametis, A., Baliakas, P., Constantinou, V., Athanasiou, E., Arsenakis, M., Anagnostopoulos, A., Papayannopoulou, T., et al. (2014). Superior long-term repopulating capacity of G-CSF+plerixafor-mobilized blood: implications for stem cell gene therapy by studies in the Hbb(th-3) mouse model. Hum. Gene Ther. Methods *25*, 317–327. https://doi.org/10.1089/hgtb.2014.005.

35. Imamura, M., Tsutsumi, Y., Miura, Y., Toubai, T., and Tanaka, J. (2003). Immune reconstitution and tolerance after allogeneic hematopoietic stem cell transplantation. Hematology *8*, 19–26. https://doi.org/10.1080/1024533031000072045.

36. Li, Z., Gilbert, J.A., Zhang, Y., Zhang, M., Qiu, Q., Ramanujan, K., Shavlakadze, T., Eash, J.K., Scaramozza, A., Goddeeris, M.M., et al. (2012). An HMGA2-IGF2BP2 axis regulates myoblast proliferation and myogenesis. Dev. Cell *23*, 1176–1188. https://doi.org/10.1016/j.devcel.2012.10.019.

37. Kessler, S.M., Pokorny, J., Zimmer, V., Laggai, S., Lammert, F., Bohle, R.M., and Kiemer, A.K. (2013). IGF2 mRNA binding protein p62/IMP2-2 in hepatocellular carcinoma: antiapoptotic action is independent of IGF2/PI3K signaling. Am. J. Physiol. Gastrointest. Liver Physiol. *304*, G328–G336. https://doi.org/10.1152/ajpgi.00005.2012.

38. Ahmed, S.M., and Dröge, P. (2019). Oncofetal HMGA2 attenuates genotoxic damage induced by topoisomerase II target compounds through the regulation of local DNA topology. Mol. Oncol. *13*, 2062–2078. https://doi.org/10.1002/1878-0261.12541.

39. Zhao, X., Peter, S., Dröge, P., and Yan, J. (2017). Oncofetal HMGA2 effectively curbs unconstrained (+) and (-) DNA supercoiling. Sci. Rep. *7*, 8440. https://doi.org/10.1038/s41598-017-09104-5.

40. Ma, Q., Ye, S., Liu, H., Zhao, Y., Mao, Y., and Zhang, W. (2024). HMGA2 promotes cancer metastasis by regulating epithelial-mesenchymal transition. Front. Oncol. *14*, 1320887. https://doi.org/10.3389/fonc.2024.1320887.

41. Moison, C., Spinella, J.F., Chagraoui, J., Lavallée, V.P., Lehnertz, B., Thiollier, C., Boivin, I., Mayotte, N., MacRae, T., Marinier, A., et al. (2022). HMGA2 expression defines a subset of human AML with immature transcriptional signature and vulnerability to G2/M inhibition. Blood Adv. *6*, 4793–4806. https://doi.org/10.1182/bloodadvances.2021005828.

42. Bai, J., Yokomizo-Nakano, T., Kubota, S., Sun, Y., Kanai, A., Iimori, M., Harada, H., Iwama, A., and Sashida, G. (2021). Overexpression of Hmga2 activates Igf2bp2 and remodels transcriptional program of Tet2-deficient stem cells in myeloid transformation. Oncogene *40*, 1531–1541. https://doi.org/10.1038/s41388-020-01629-w.

43. Yang, S., Gu, Y., Wang, G., Hu, Q., Chen, S., Wang, Y., and Zhao, M. (2019). HMGA2 regulates acute myeloid leukemia progression and sensitivity to daunorubicin via Wnt/beta-catenin signaling. Int. J. Mol. Med. *44*, 427–436. https://doi.org/10.3892/ijmm.2019.4229.

44. De Martino, M., Esposito, F., and Fusco, A. (2022). Critical role of the high mobility group A proteins in hematological malignancies. Hematol. Oncol. *40*, 2–10. https://doi.org/10.1002/hon.2934.

45. De Ravin, S.S., Liu, S., Sweeney, C.L., Brault, J., Whiting-Theobald, N., Ma, M., Liu, T., Choi, U., Lee, J., O'Brien, S.A., et al. (2022). Lentivector cryptic splicing mediates increase in CD34+ clones expressing truncated HMGA2 in human X-linked severe combined immunodeficiency. Nat. Commun. *13*, 3710. https://doi.org/10.1038/s41467-022-31344-x.

46. Wang, G.P., Ciuffi, A., Leipzig, J., Berry, C.C., and Bushman, F.D. (2007). HIV integration site selection: analysis by massively parallel pyrosequencing reveals association with epigenetic modifications. Genome Res. *17*, 1186–1194. https://doi.org/10.1101/gr.6286907.

47. Ciuffi, A. (2008). Mechanisms governing lentivirus integration site selection. Curr. Gene Ther. *8*, 419–429. https://doi.org/10.2174/156652308786848021.

48. Trobridge, G., Beard, B.C., and Kiem, H.P. (2005). Hematopoietic stem cell transduction and amplification in large animal models. Hum. Gene Ther. *16*, 1355–1366. https://doi.org/10.1089/hum.2005.16.1355.

49. Allay, J.A., Persons, D.A., Galipeau, J., Riberdy, J.M., Ashmun, R.A., Blakley, R.L., and Sorrentino, B.P. (1998). In vivo selection of retrovirally transduced hematopoietic stem cells. Nat. Med. *4*, 1136–1143. https://doi.org/10.1038/2632.

50. Baum, C., Eckert, H.G., Stockschläder, M., Just, U., Hegewisch-Becker, S., Hildinger, M., Uhde, A., John, J., and Ostertag, W. (1996). Improved retroviral vectors for hematopoietic stem cell protection and *in vivo* selection. J. Hematother. *5*, 323–329. https://doi.org/10.1089/scd.1.1996.5.323.

51. Guo, Q., Zhang, J., Parikh, K., Brinkley, A., Lin, S., Zakarian, C., Pernet, O., Shimizu, S., Khamaikawin, W., Hacke, K., et al. (2024). In vivo selection of anti-HIV-1 gene-modified human hematopoietic stem/progenitor cells to enhance engraftment and HIV-1 inhibition. Mol. Ther. *32*, 384–394. https://doi.org/10.1016/j.ymthe.2023.12.007.

52. Choudhary, R., Baturin, D., Fosmire, S., Freed, B., and Porter, C.C. (2013). Knockdown of HPRT for selection of genetically modified human hematopoietic progenitor cells. PLoS One *8*, e59594. https://doi.org/10.1371/journal.pone.0059594.

53. Marone, R., Landmann, E., Devaux, A., Lepore, R., Seyres, D., Zuin, J., Burgold, T., Engdahl, C., Capoferri, G., Dell'Aglio, A., et al. (2023). Epitope-engineered human hematopoietic stem cells are shielded from CD123-targeted immunotherapy. J. Exp. Med. *220*, e20231235. https://doi.org/10.1084/jem.20231235.

54. Casirati, G., Cosentino, A., Mucci, A., Salah Mahmoud, M., Ugarte Zabala, I., Zeng, J., Ficarro, S.B., Klatt, D., Brendel, C., Rambaldi, A., et al. (2023). Epitope editing enables targeted immunotherapy of acute myeloid leukaemia. Nature *621*, 404–414. https://doi.org/10.1038/s41586-023-06496-5.

55. Wellhausen, N., O'Connell, R.P., Lesch, S., Engel, N.W., Rennels, A.K., Gonzales, D., Herbst, F., Young, R.M., Garcia, K.C., Weiner, D., et al. (2023). Epitope base editing CD45 in hematopoietic cells enables universal blood cancer immune therapy. Sci. Transl. Med. *15*, eadi1145. https://doi.org/10.1126/scitranslmed.adi1145.

56. Watts, K.L., Beard, B.C., Wood, B.L., Trobridge, G.D., Humphries, R.K., Adams, A.B., Nelson, V., and Kiem, H.P. (2014). No evidence of clonal dominance after transplant

of HOXB4-expanded cord blood cells in a nonhuman primate model. Exp. Hematol. *42*, 497–504. https://doi.org/10.1016/j.exphem.2014.03.007.

57. de la Chapelle, A., Träskelin, A.L., and Juvonen, E. (1993). Truncated erythropoietin receptor causes dominantly inherited benign human erythrocytosis. Proc. Natl. Acad. Sci. USA *90*, 4495–4499. https://doi.org/10.1073/pnas.90.10.4495.

58. Sokol, L., Luhovy, M., Guan, Y., Prchal, J.F., Semenza, G.L., and Prchal, J.T. (1995). Primary familial polycythemia: a frameshift mutation in the erythropoietin receptor gene and increased sensitivity of erythroid progenitors to erythropoietin. Blood *86*, 15–22.

59. Uchida, N., Drysdale, C., Yapundich, M., Gamer, J., Nassehi, T., Wielgosz, M., Ferrara, F., Haro-Mora, J.J., Demirci, S., Bonifacino, A., et al. (2019). Truncated erythropoietin receptors confer an *in vivo* selective advantage in gene-modified erythroid cells expressing fetal hemoglobin due to bcl11a interference. Blood *134*, 2063. abstract 801.

60. Roman-Rodriguez, F.J., Ugalde, L., Alvarez, L., Diez, B., Ramirez, M.J., Risueno, C., Corton, M., Bogliolo, M., Bernal, S., March, F., et al. (2019). NHEJ-Mediated Repair of CRISPR-Cas9-Induced DNA Breaks Efficiently Corrects Mutations in HSPCs from Patients with Fanconi Anemia. Cell Stem Cell *25*, 607–621.e7. https://doi.org/10.1016/j.stem.2019.08.016.

61. Rio, P., Navarro, S., Guenechea, G., Sanchez-Dominguez, R., Lamana, M.L., Yanez, R., Casado, J.A., Mehta, P.A., Pujol, M.R., Surralles, J., et al. (2017). Engraftment and *in vivo* proliferation advantage of gene-corrected mobilized CD34(+) cells from Fanconi anemia patients. Blood *130*, 1535–1542. https://doi.org/10.1182/blood-2017-03-774174.

62. Fitzhugh, C.D., Hsieh, M.M., Bolan, C.D., Saenz, C., and Tisdale, J.F. (2009). Granulocyte colony-stimulating factor (G-CSF) administration in individuals with sickle cell disease: time for a moratorium? Cytotherapy *11*, 464–471. https://doi.org/10.1080/14653240902849788.

63. Li, C., Goncalves, K.A., Raskó, T., Pande, A., Gil, S., Liu, Z., Izsvák, Z., Papayannopoulou, T., Davis, J.C., Kiem, H.P., and Lieber, A. (2021). Single-dose MGTA-145/plerixafor leads to efficient mobilization and *in vivo* transduction of HSCs with thalassemia correction in mice. Blood Adv. *5*, 1239–1249. https://doi.org/10.1182/bloodadvances.2020003714.

64. Li, C., Anderson, A.K., Ruminski, P., Rettig, M., Karpova, D., Kiem, H.P., Dipersio, J.F., and Lieber, A. (2024). A simplified, G-CSF-free mobilization procudure allows for *in vivo* HSC gene therapy of sickle cell disease in a mouse model. Blood Adv *8*, 4089–4101.

65. Wang, H., Georgakopoulou, A., Psatha, N., Li, C., Capsali, C., Samal, H.B., Anagnostopoulos, A., Ehrhardt, A., Izsvák, Z., Papayannopoulou, T., et al. (2019). In vivo hematopoietic stem cell gene therapy ameliorates murine thalassemia intermedia. J. Clin. Invest. *129*, 598–615. https://doi.org/10.1172/JCI122836.

66. Wang, H., Germond, A., Li, C., Gil, S., Kim, J., Kiem, H.P., and Lieber, A. (2022). In vivo HSC transduction in rhesus macaques with an HDAd5/3+ vector targeting desmoglein 2 and transiently overexpressing cxcr4. Blood Adv. *6*, 4360–4372. https://doi.org/10.1182/bloodadvances.2022007975.

67. Wang, H., Shayakhmetov, D.M., Leege, T., Harkey, M., Li, Q., Papayannopoulou, T., Stamatoyannopolous, G., and Lieber, A. (2005). A capsid-modified helper-dependent adenovirus vector containing the beta-globin locus control region displays a nonrandom integration pattern and allows stable, erythroid-specific gene expression. J. Virol. *79*, 10999–11013. https://doi.org/10.1128/JVI.79.17.10999-11013.2005.

68. Li, H., Handsaker, B., Wysoker, A., Fennell, T., Ruan, J., Homer, N., Marth, G., Abecasis, G., and Durbin, R.; 1000 Genome Project Data Processing Subgroup (2009). The Sequence Alignment/Map format and SAMtools. Bioinformatics *25*, 2078–2079. https://doi.org/10.1093/bioinformatics/btp352.

69. Quinlan, A.R., and Hall, I.M. (2010). BEDTools: a flexible suite of utilities for comparing genomic features. Bioinformatics *26*, 841–842. https://doi.org/10.1093/bioinformatics/btq033.

70. Yu, G., Wang, L.G., and He, Q.Y. (2015). ChIPseeker: an R/Bioconductor package for ChIP peak annotation, comparison and visualization. Bioinformatics *31*, 2382–2383. https://doi.org/10.1093/bioinformatics/btv145.

71. Lawrence, M., Huber, W., Pagès, H., Aboyoun, P., Carlson, M., Gentleman, R., Morgan, M.T., and Carey, V.J. (2013). Software for computing and annotating genomic ranges. PLoS Comput. Biol. *9*, e1003118. https://doi.org/10.1371/journal.pcbi.1003118.

72. Wagih, O. (2017). ggseqlogo: a versatile R package for drawing sequence logos. Bioinformatics *33*, 3645–3647. https://doi.org/10.1093/bioinformatics/btx469.

73. Gel, B., and Serra, E. (2017). karyoploteR: an R/Bioconductor package to plot customizable genomes displaying arbitrary data. Bioinformatics *33*, 3088–3090. https://doi.org/10.1093/bioinformatics/btx346.

74. McKenna, A., Hanna, M., Banks, E., Sivachenko, A., Cibulskis, K., Kernytsky, A., Garimella, K., Altshuler, D., Gabriel, S., Daly, M., and DePristo, M.A. (2010). The Genome Analysis Toolkit: a MapReduce framework for analyzing next-generation DNA sequencing data. Genome Res. *20*, 1297–1303. https://doi.org/10.1101/gr.107524.110.

75. Wang, K., Li, M., and Hakonarson, H. (2010). ANNOVAR: functional annotation of genetic variants from high-throughput sequencing data. Nucleic Acids Res. *38*, e164. https://doi.org/10.1093/nar/gkq603.

OMTM, Volume *32*

Supplemental information

Auto-expansion of *in vivo* HDAd-transduced hematopoietic stem cells by constitutive expression of tHMGA2

Hongjie Wang, Aphrodite Georgakopoulou, Evangelos Nizamis, Ka Wai Mok, Raïssa Eluère, Robert A. Policastro, Paul N. Valdmanis, and André Lieber

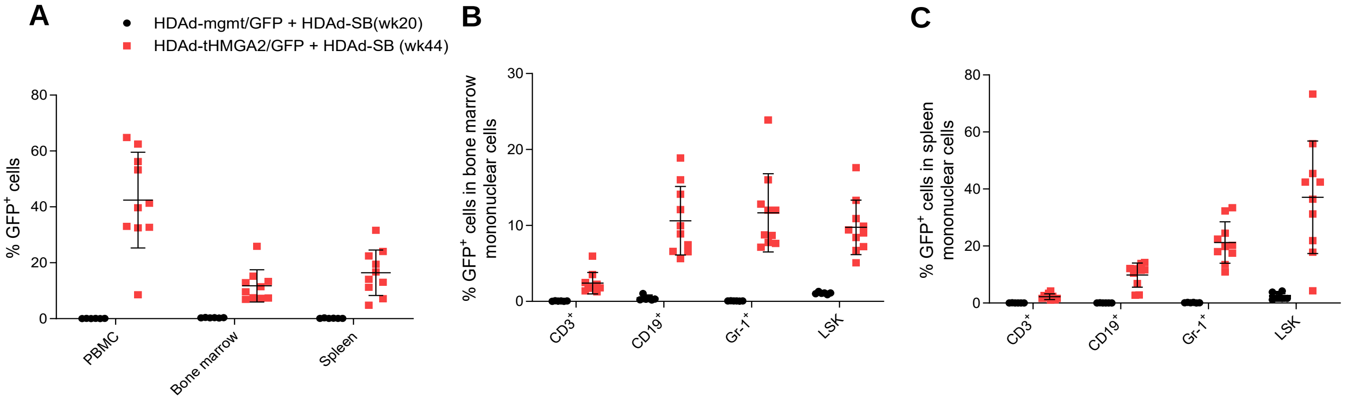

Fig.S1. *In vivo* transduction with HDAd-mgmt/GFP + HDAd-SB and HDAd-tHMGA2/GFP + HDAd-SB- Analysis of PBMC, bone marrow, and spleen cells. A) The percentage of GFP-positive cells in total PBMC, bone marrow and spleen mononuclear cells. **B) and C)** Percentage of GFP-positive within lineage-positive cells and LSK cells within bone marrow and spleen. Each symbol is an individual animal. Mean and error bars (+/-SEM) are shown.

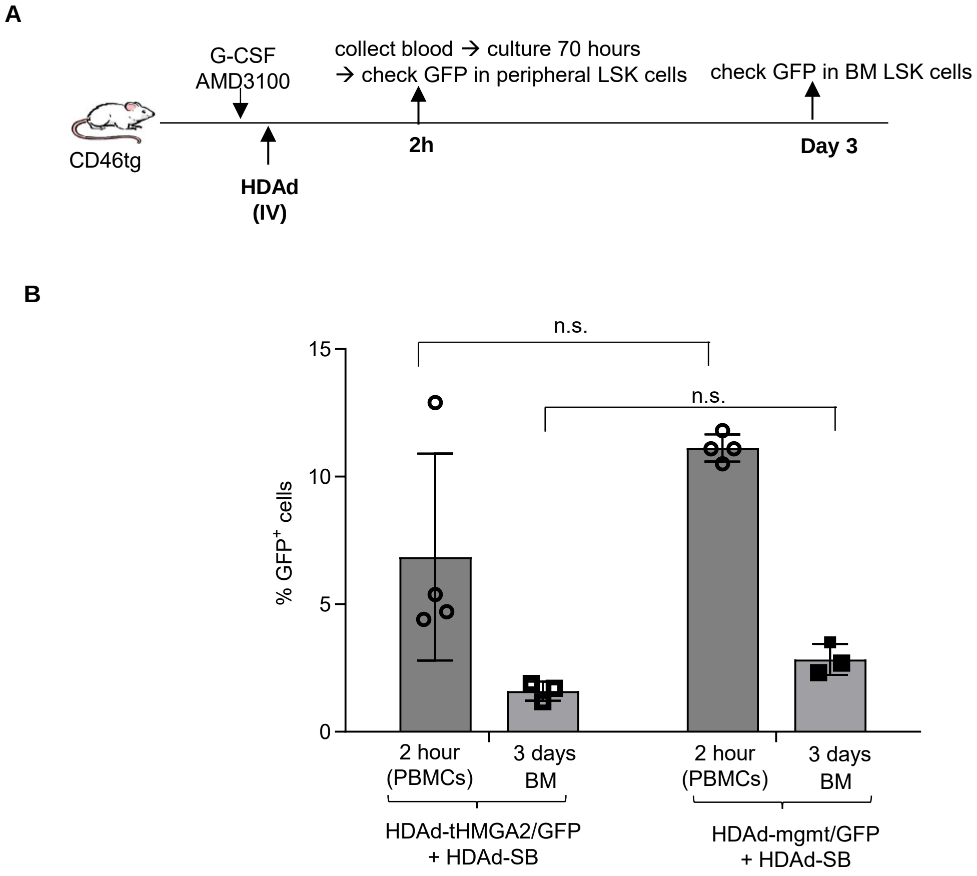

Fig.S2. Initial *in vivo* transduction of mobilized HSCs. A) Schematic of experiment. HSCs in hCD46tg mice were mobilized by G-CSF for 4 days, followed by a single injection of AMD3100 on day 5. Thirty and 60 minutes after AMD3100, animals were intravenously injected with HDAd vectors through the retro-orbital plexus ($4x10^{10}$ viral particles per injection per mouse; same dose as in Fig.1). Transduction of mobilized, peripheral LSK cells was analyzed by harvesting PBMCs at 2 hours after the last virus injection, and culturing them for 3 days to allow for GFP expression. At day after HDAd injection, animals were sacrificed and the percentage of GFP^+ LSK cells in the bone marrow was measured. **B)** Initial transduction of LSK cells. Shown are percentages of GFP^+ LSK cells after injection of HDAd-tHMGA2/GFP + HDAd-SB and HDAd-mgmt/GFP + HDAd-SB in (mobilized) peripheral blood LSK cells (at 2 hours) and in LSK cells that returned to the bone marrow (day 3). Each symbol is an individual animal.

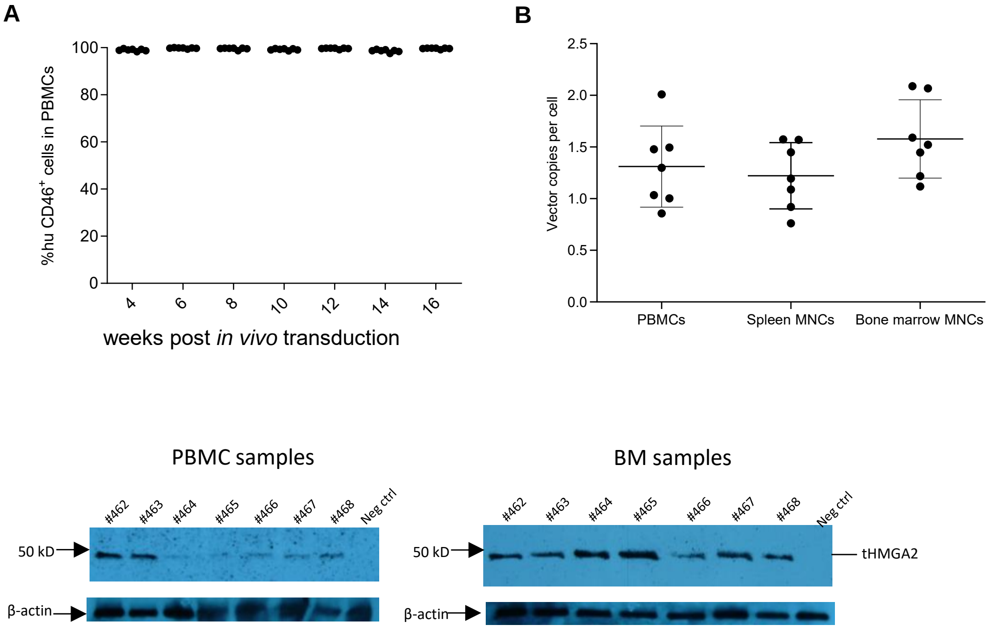

Fig.S3 Analysis of secondary recipients transplanted with Lin^{-} cells from HDAd-tHMGA2+HDAdSB transduced mice. A) Engraftment based on the percentage of human CD46-positive cells in PBMCs. **B)** VCN per cell in PBMCs, BM MNCs and spleen MNCs of secondary recipients at week 18 after transplantation. Data are shown as means ± SEM. **C)** Western blot showing the expression of tHMGA2 protein in PBMCs and bone marrow MNCs at week 18 after transplantation.

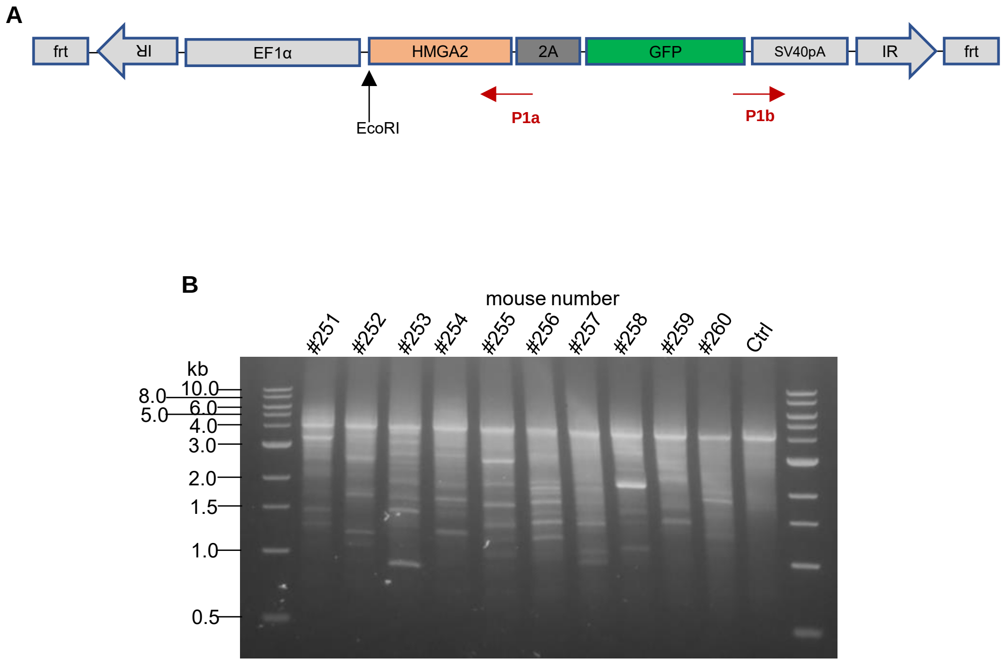

Fig.S4 Integration analysis by inverse PCR. A) The transposon (between two IR elements) in the HDAd vectors are integrated into chromosomal DNA. Genomic DNA was digested with EcoRI, which cuts inside the transposon and re-ligated. PCR were performed with two vector specific primers (P1a, P1b)to amplify transposon/chromosomal DNA junctions. **B)** Agarose gel electrophoresis showing PCR products. Note that the vector specific primer set used has an unspecific background band (~4.0 kb) in all mice including untransduced control mice.

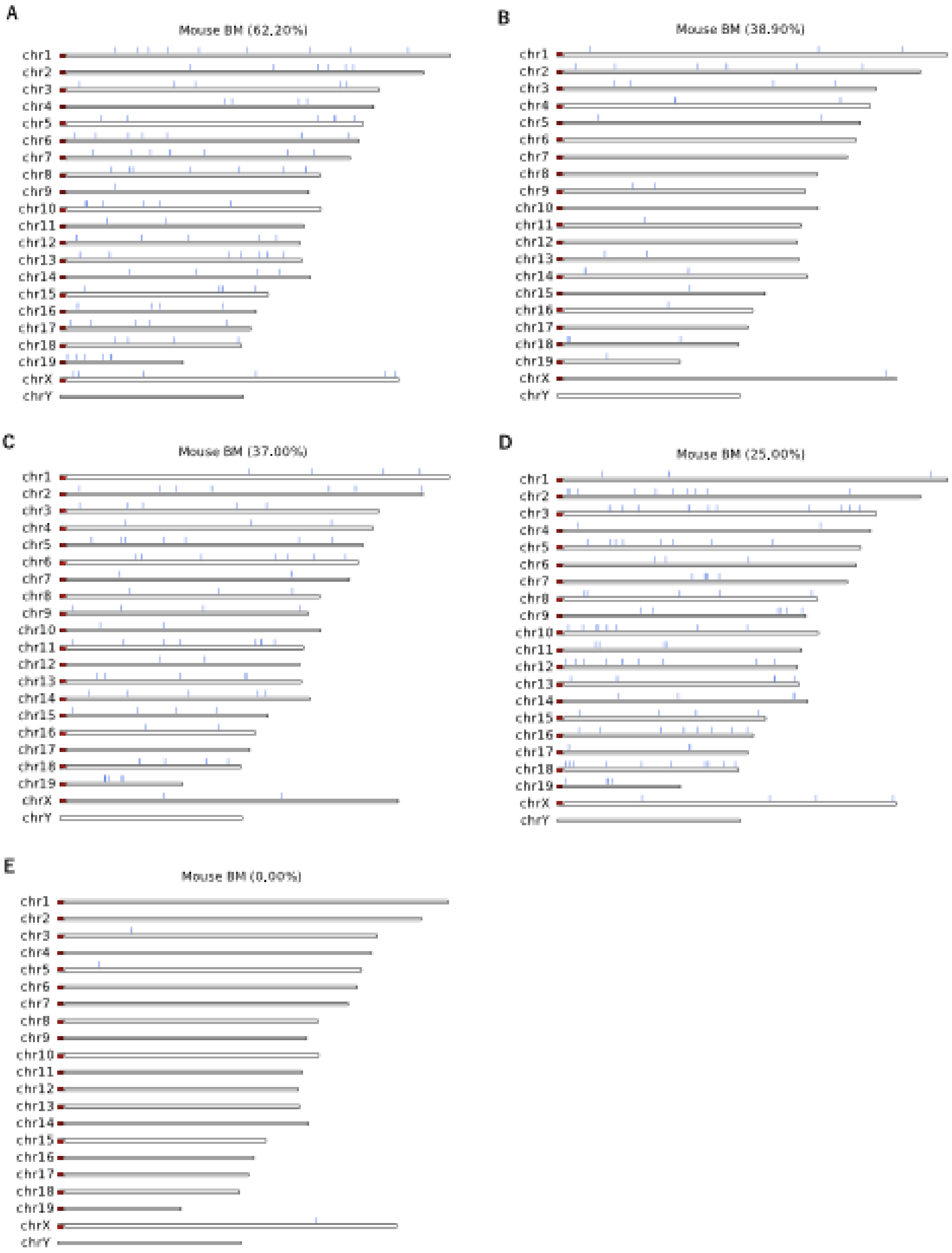

Fig.S5 Chromosomal distribution of integration sites. Chr

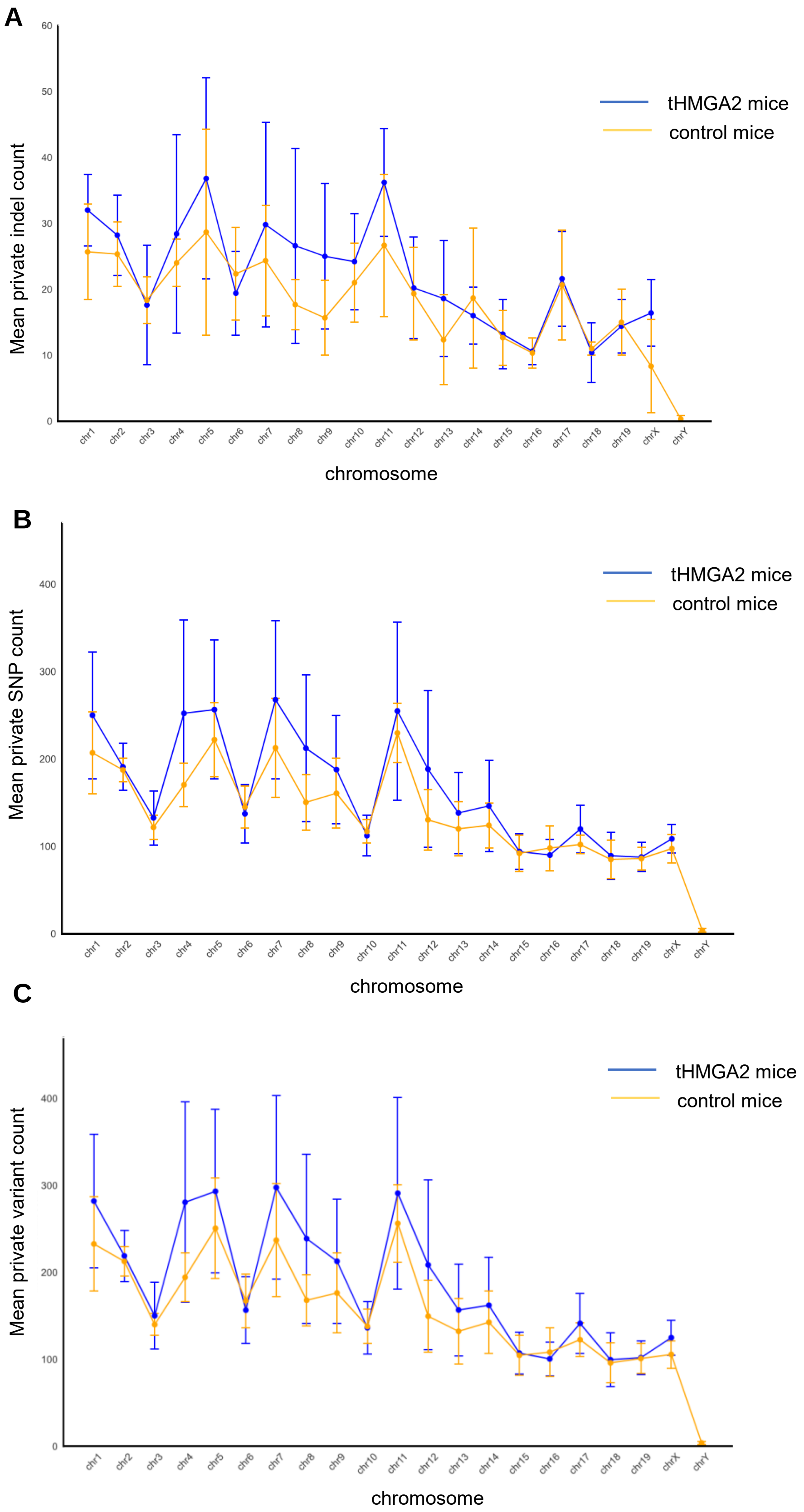

Fig.S6 Lineplot with error bars showing standard deviation of values for private variants of HDAd-tHMGA2 + HDAd-SB compared with untreated control mice. Private variants (SNPs/Indels) were categorized by chromosome and compared between HDAd-tHMGA2 + HDAd-SB treated mice (BM1-BM-5) compared with untreated control mice (N1-N3). Mean values and standard deviations were calculated across samples for each chromosome. Private variants are unique to each sample, meaning they are not found in any other treated or control samples. They were calculated for **A)** All Variants**, B)** Indels, and **C)** SNPs.

TableS1 TRACE sequencing primers. The oligo sequences used for tagmentation and PCR in TRACE sequencing.

Oligo	Sequence
Tn5 adaptor top	GACCCGGGAGATCTGAATTCAGTGGCACAGCAGTTAGGNNNNNNNNAGATGTGTATAAGAGACAG
Tn5 adaptor bottom	Phos-CTGTCTCTTATACACATC/invT
Tn1c (PCR #1 F)	CTTGTGTCATGCACAAAGTAGATGTCC
LCI (PCR #1 R)	GACCCGGGAGATCTGAATTC
MA-F (PCR #2 F)	ACACTCTTTCCCTACACGACGCTCTTCCGATCTNNNNCGAGTTTTAATGACTCCAACT
MA-LCII (PCR #2 R)	GTGACTGGAGTTCAGACGTGTGCTCTTCCGATCTAGTGGCACAGCAGTTAGG

TableS2 Genome-wide insertion locations (provided as Excel file)

TableS3 Statistics of the coverage on target exon regions

[Target] Target Reads	237195756
[Target] Target Data (Mb)	27023.94
[Target] Len of region	49387927
[Target] Average depth	547.18
[Target] Coverage (>0x)	99.90%
[Target] Coverage (>/=10x)	99.61%
[Target] Coverage (>/=30x)	98.72%
[Target] Coverage (>/=100x)	94.34%
[flank] flank size	200
[flank] Len of region	118267836
[flank] Average depth	318.75
[flank] flank reads	254868740
[flank] flank Data (Mb)	37697.90
[flank] Coverage (>0x)	99.53%
[flank] Coverage (>/=10x)	95.37%
[flank] Coverage (>/=30x)	85.96%
[flank] Coverage (>/=100x)	66.71%
